# The molecular basis of lamin-specific chromatin interactions

**DOI:** 10.1038/s41594-025-01622-5

**Published:** 2025-08-01

**Authors:** Baihui Wang, Rafael Kronenberg-Tenga, Valentina Rosti, Emanuele Di Patrizio Soldateschi, Qiang Luo, Ugo Maria Iannacchero, Louise Pinet, Matthias Eibauer, Rajaa Boujemaa-Paterski, Benjamin Schuler, Chiara Lanzuolo, Ohad Medalia

**Affiliations:** 1 Department of Biochemistry, University of Zurich, Zurich, Switzerland; 2 Istituto Nazionale Genetica Molecolare ‘Romeo ed Enrica Invernizzi’, Milan, Italy; 3 Institute of Biomedical Technologies, National Research Council, Milan, Italy

**Keywords:** Cryoelectron microscopy, Cryoelectron tomography, Nuclear organization, Chromosomes

## Abstract

In the cell nucleus, chromatin is anchored to the nuclear lamina, a network of lamin filaments and binding proteins that underly the inner nuclear membrane. The nuclear lamina is involved in chromatin organization through the interaction of lamina-associated domains within the densely packed heterochromatin regions. Using cryo-focused ion beam milling in conjunction with cryo-electron tomography, we analyzed the distribution of nucleosomes at the lamin–chromatin interface at the nanometer scale. Depletion of lamins A and C reduced nucleosome concentration at the nuclear periphery, while B-type lamin depletion contributed to nucleosome density in proximity to the lamina but not further away. We then investigated whether specific lamins can mediate direct interactions with chromatin. Using cryo-electron microscopy, we identified a specific binding motif of the lamin A tail domain that interacts with nucleosomes, distinguishing it from the other lamin isoforms. Furthermore, we examined chromatin structure dynamics using a genome-wide analysis that revealed lamin-dependent macroscopic-scale alterations in gene expression and chromatin remodeling. Our findings provide detailed insights into the dynamic and structural interplay between lamin isoforms and chromatin, molecular interactions that shape chromatin architecture and epigenetic regulation.

## Abstract (web summary)

Wang, Kronenberg-Tenga, Rosti and colleagues use several structural approaches to analyze the distribution of nucleosomes at the lamin–chromatin interface, test the impact of lamins on nucleosome density and identify a lamin A nucleosome-binding motif.

## Main

Nuclear lamins provide mechanical support and safeguard the integrity of the cell nucleus. At the nuclear lamina (NL), lamins contribute to the organization of chromatin^1,2^. In mammals, four main lamin isoforms are expressed: the A-type lamins (A and C; A/C) and the B-type lamins (B1 and B2; B1/B2)^3^. During their posttranslational processing, lamins A, B1 and B2 are farnesylated at their C terminus. Notably, while lamin A loses its farnesyl group through a subsequent cleavage, the B-type lamins retain their farnesylation, serving as anchors to the inner nuclear membrane (INM)^4^. The two types of lamin proteins have been described to form two separate but interconnected filamentous meshworks^5^, wherein the B-type lamin meshwork is localized closer to the nuclear membrane^6,7^. Previous investigations of the NL of mouse embryonic fibroblasts (MEFs) showed a 14–16-nm-thick meshwork consisting of ~3.5-nm-thick lamin filaments^8,9^.

Knockout (KO) of A-type lamins in MEFs greatly impacts nuclear mechanics by reducing stiffness and structural integrity^10–12^ and decoupling nuclear and cytoplasmic forces^13^. These observations suggest direct interactions between lamins A/C and chromatin^14^ at the docking sites of heterochromatic regions^15–18^. These large sites, commonly termed lamina-associated domains (LADs), can extend up to 10 Mb in length and are typically enriched with the H3K9me3 repressive mark, rendering them transcriptionally inactive. However, 10% of the genes inside LADs are still expressed^19^. Interestingly, A-type lamins were shown to also interact with euchromatic DNA^20^ and with facultative H3K27me3-enriched chromatin^21–24^, whereas lamin B1 is also localized to open and actively expressed chromatin regions^25^. Insufficient lamin expression results in genome remodeling, manifested by alterations in histone markers and the detachment of LADs^26,27^. Genome-wide approaches such as HiC or DamID have detected lamin-dependent chromatin alterations^17,26,27^; however, the direct molecular interactions and effects of the lamin isoforms on chromatin organization remain elusive.

## Results

### Chromatin and lamin organization at the nuclear envelope imaged by cryo-electron tomography

**Fig. 1:**
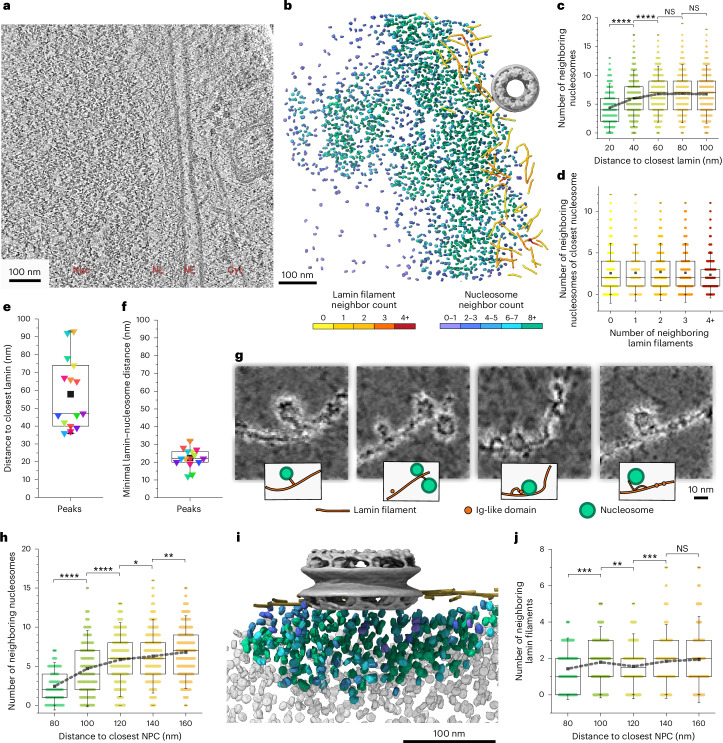
The local concentration and distribution of nucleosomes at the NE. **a**, An *x*–*y* tomographic slice, 8.8 Å in thickness, of an MEF cell thinned by cryo-FIB. The nucleoplasm (Nuc), NL, NE and cytoplasm (Cyt) are indicated. **b**, Segmented view of a tomogram depicting the NE of a cell. Nucleosomes and lamins are colored on the basis of their local concentration, from purple to green and yellow to red. The NPC was manually placed (gray, EMD-12814). **c**, The local concentration of nucleosomes was defined as the number of neighboring nucleosomes within a 24-nm radius, plotted as a function of their distance from the NL. The concentration plateaued at an average of seven neighbouring nucleosomes (~1.4 × 10⁵ nucleosomes per μm³) beyond 60 nm from the NL. **d**, The relationship between nucleosome concentration and lamin concentration is shown. For each lamin segment, the number of neighboring lamin filaments was determined and plotted against the local nucleosome concentration of its nearest nucleosome. **e**, The nucleosome concentration probability was plotted as a function of the distance to the closest lamin filament in each tomogram (Extended Data Fig. 4g). Their associated distance of peaks is plotted in **e**, showing a median value of 47 nm. **f**, The nucleosome probability density as a function of the distance between nucleosomes and lamins is plotted for each tomogram (Extended Data Fig. 4i). The peaks are plotted against associated distance values in **f**. The median distance was 22 ± 5 nm. **g**, Images highlighting examples of direct lamin–nucleosome interactions. **h**, The nucleosome concentration is plotted against the distance to the center of the closest NPC. **i**, Segmented view of a measurement in **h**. The nucleosomes are colored on the basis of their concentration. **j**, The concentration of lamin filaments is plotted against their distance to the closest NPC. All box plots show a box between the 25th and 75th percentiles, the median as a horizontal line, the mean as a black square and 1.5 s.d. as whiskers. Significance was calculated using a one-way ANOVA. ****P < 0.0001, ***P < 0.001, **P < 0.01 and *P < 0.05; not significant (NS), *P* > 0.05. A total of 15 tomograms were used for the analysis shown in **c–f**, **h** and **j**. Gray dashed lines in **c**, **h** and **j** connect the mean values of each column. Each colored triangle in **e** and **f** represents the most frequent minimal distance between nucleosomes and lamin filaments, in each tomogram.

**Extended Data Fig. 1:**
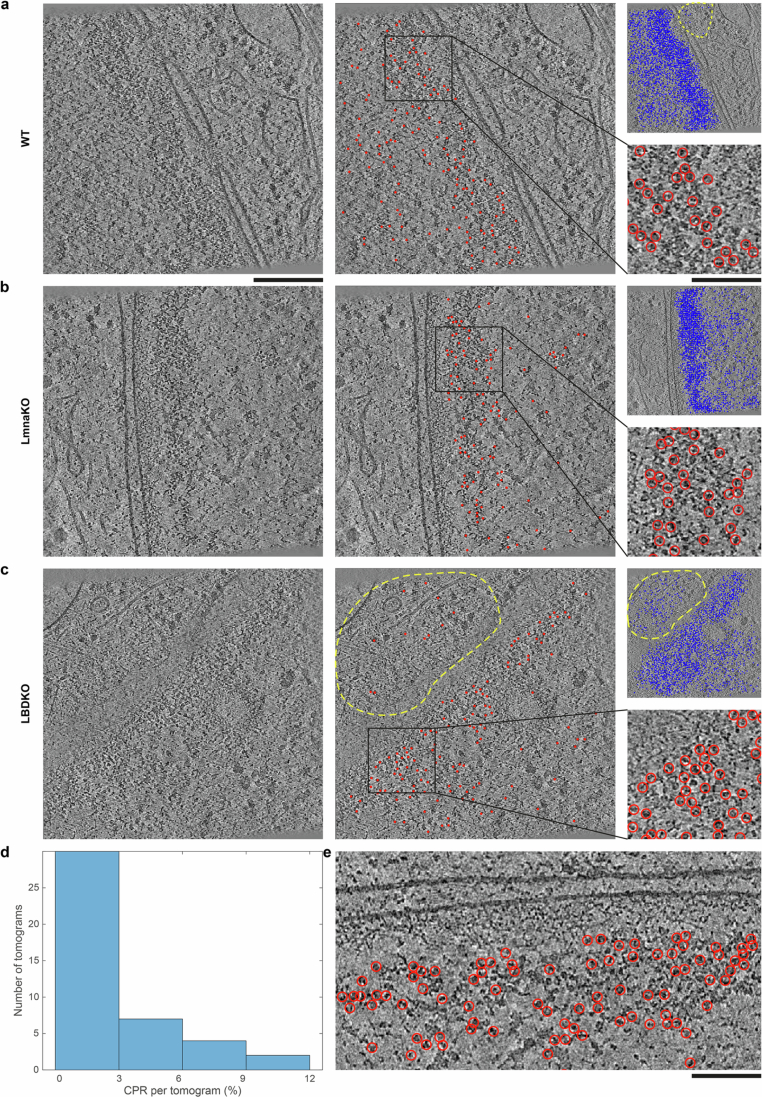
Detection of nucleosomes in tomograms of cryo-FIB milled lamellae of MEFs. **a**. Nucleosome picking is shown for one of the WT tomograms. The image on the left shows a xy slice with 8.8 Å thickness through the tomogram. The nuclear envelope was imaged in a side view orientation. Scale bar is 200 nm. The image in the middle shows the same xy-slice as on the left, together with the coordinates of the nucleosomes (red dots), which were detected in that slice. The apparent low density of picked nucleosomes in this visualization is due to the fact that only nucleosomes with their z coordinate falling exactly within this xy-slice are displayed. The top image on the right side shows this xy-slice, overlaid with all the nucleosome coordinates (blue dots), which were picked in this tomogram. This context was used to annotate cytoplasmic false-positive nucleosome detections (blue dots encircled with a yellow dashed line). In this tomogram, 10 cytoplasmic false-positive particles were detected. A total of 2673 subtomograms were extracted from this tomogram. Therefore, the individual cytoplasmic (false-positive) picking ratio (CPR) of this tomogram is 0.4%. The bottom image on the right side shows a zoomed view on the picked nucleosomes (red circles). Scale bar is 100 nm. **b**. Nucleosome picking is shown for one of the LmnaKO tomograms. In this tomogram zero cytoplasmic false-positive particles were detected, therefore the CPR is 0%. **c**. Nucleosome picking is shown for one of the LBDKO tomograms. Here, the nuclear envelope was imaged in a top view orientation. Cytoplasmic false-positive particles are shown per xy-slice in the middle image (red dots encircled with a yellow dashed line) and all cytoplasmic false-positive particles identified in this tomogram are shown in the top image on the right (blue dots encircled with a yellow dashed line). In this tomogram, 156 cytoplasmic false-positive particles were detected. A total of 1842 subtomograms were extracted from this tomogram and therefore its CPR is 8.5%. **d**. Histogram of all CPR values per tomogram of the complete dataset (43 tomograms). Most of the tomograms (30 tomograms) exhibit CPR values between 0 to 3%. **e**. Zoomed view on individual nucleosome detections. The nucleosome coordinates, which were picked in the shown xy-slice, are depicted as red circles. Scale bar is 100 nm.

**Extended Data Fig. 2:**
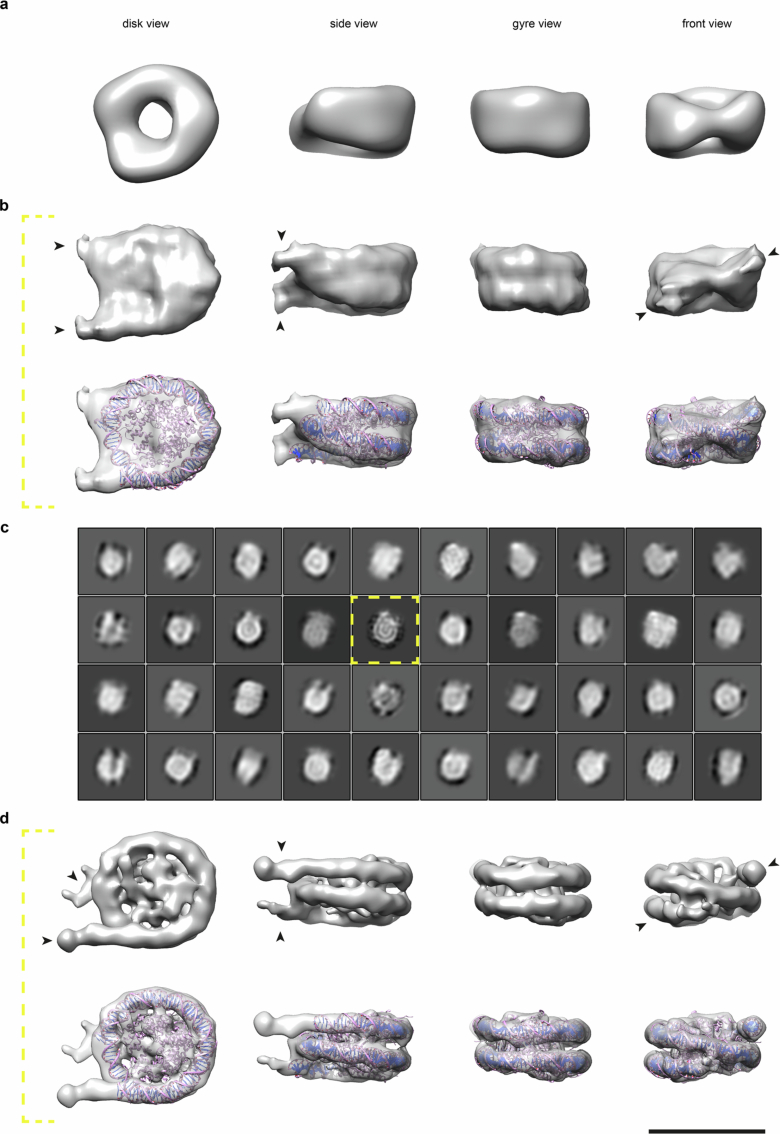
Sub-tomogram averaging of in-situ nucleosomes. **a**. The initial reference for 3D refinement and 3D classification is shown in 4 different views, namely disk view, side view, gyre view, and front view. The reference was obtained by low-pass filtering the nucleosome structure EMD 33132 to 40 Å resolution. At this resolution the histones in the core of the nucleosome disappear and the DNA resembles a torus. **b**. In the top row the in-situ nucleosome consensus average is displayed. Compared to the initial reference, the density of the histone core is restored and linker DNA densities appear (black arrowheads). In the bottom row the docking of the nucleosome model PDB-7XD1 in the consensus nucleosome average is shown. **c**. The subtomograms, which were averaged to form the consensus average (103173 particles), were classified with 3D classification into 40 classes. The image shows the central xy-slices (thickness 4.4 Å) of the resulting class averages in disk view orientation. All class averages resemble nucleosome features at varying resolutions. The class average, which attracted the most particles (13255 particles), is framed by a yellow dashed line. **d**. Subsequently, these particles were averaged with 3D refinement. In the top row the resulting average is displayed. It shows clear features of a canonical nucleosome structure, with extended linker DNA (black arrowheads), a well-defined histone core, and the DNA wrapped around the histone core in a left-handed manner. In the bottom row the docking of the nucleosome model PDB-7XD1 in the canonical nucleosome average is shown. Scale bar is 10 nm.

**Extended Data Fig. 3:**
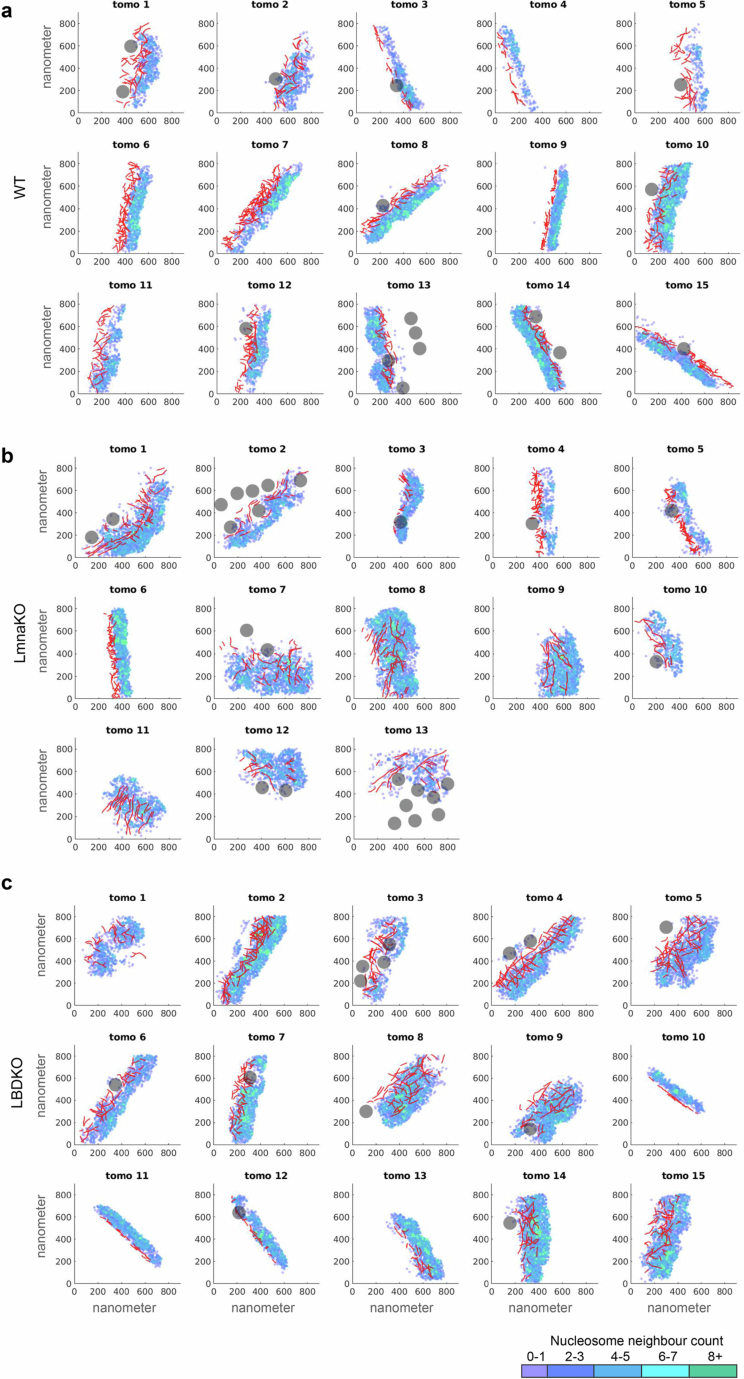
Gallery of tomograms used for the analysis of WT (a), LmnaKO (b), and LBDKO (c) MEFs. Each tomogram is depicted by the xy-coordinates of the three analysed structures: lamin coordinates are shown in red, nucleosome coordinates are coloured by increasing concentration, as indicated by the calibration bar, and NPC coordinates are shown in grey.

**Extended Data Fig. 4:**
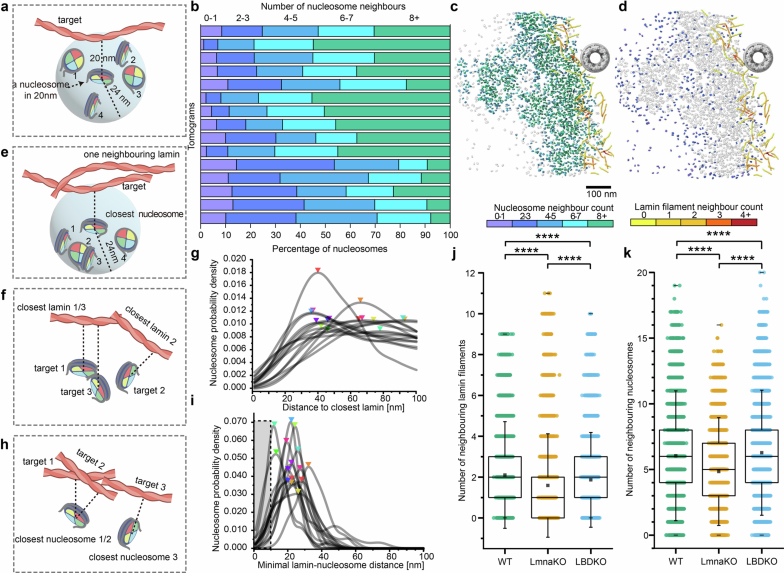
Local distribution and concentration of nucleosomes and lamins in WT, LmnaKO, LBDKO MEF cells. **a**. A schematic illustration indicating how we quantified local nucleosome concentration as a function of distance from a lamin filament. The number of nucleosomes located within a 24 nm radius of a nucleosome positioned 20 nm from a lamin filament. **b**. Bar plot showed nucleosome concentration in each tomogram from WT MEFs. **c-d**. Regions with high local nucleosome concentration, with ≥4 neighbouring nucleosomes (**c**) or nucleosomes with ≤3 or fewer neighbours in grey (**d**). **e**. A Schematic illustration exemplifying the approach used to measure the relationship between local nucleosome concentration and lamin concentration. For each lamin segment, the number of neighbouring lamin segments within a 24 nm radius was quantified. The coordinates of the nearest nucleosome to each lamin segment were then identified, and the number of neighbouring nucleosomes within a 24 nm radius of that nucleosome was calculated. These values were plotted as a function of the corresponding local lamin density. **f**. A schematic diagram illustrating how the distance from each nucleosome to its nearest lamin filament was measured. These distances were plotted in (**g**) for each tomogram, using a line fit for an average of ~2400 particles per tomogram. High-density peaks are indicated by triangles. **h**. A schematic diagram illustrating how the distance from each lamin filament to its nearest nucleosome was determined. These distances were plotted in (**i**) for each tomogram. The peaks are indicated by triangles. The shadowed region in (**i**) highlighted that 1.6% of nucleosomes are located within 10 nm of lamin filaments. In these instances, direct lamin-nucleosome interactions can be detected. **j**. For each lamin segment, the number of neighbouring filaments was plotted for each cell line. The mean neighbouring filaments count was 2.1, 1.6 and 1.9 for WT, LmnaKO and LBDKO cells, respectively. **k**. The number of neighbouring nucleosomes for each nucleosome was plotted, resulting in mean values of 6.0, 4.8 and 6.3 neighbouring nucleosomes for WT, LmnaKO and LBDKO cells, respectively. All boxplots show a box between 25th and 75th percentile, median as a horizontal line, mean as a black square, whiskers represent 1.5 standard deviations. Significance calculated using a one-way ANOVA. p-value < 0.0001 (****).

To dissect the contribution of nuclear lamins to the organization of the nuclear envelope (NE), we analyzed tomograms of cryo-focused ion beam (cryo-FIB)-milled, vitrified cells (Methods) in which the cytoplasm, NE, NL and nucleosomes were clearly observable (Fig. 1a). Next, the coordinates of nuclear lamins, nuclear pore complexes (NPCs) and nucleosomes were extracted from each nuclear volume (Fig. 1b, Extended Data Fig. 1 and Supplementary Videos 1–3). Subsequently, the nucleosomes were subjected to subtomogram averaging and classification^28,29^, yielding a 13-Å-resolved structure exhibiting the typical appearance in which a DNA double strand is wrapped around a histone octamer (Extended Data Fig. 2, Supplementary Fig. 1 and Table 1). We then measured the in situ local concentrations and distributions of lamins and nucleosomes within LAD regions (Fig. 1 and Extended Data Fig. 3), where chromatin is attached to the NL; these regions are commonly attributed as heterochromatin-rich domains^30^. We quantified the number of neighboring nucleosomes within a distance of 24 nm for each nucleosome, reflecting the local nucleosome concentration (Methods and Extended Data Fig. 4a). Our analysis resolved varying nucleosome concentrations within these regions (Fig. 1b and Extended Data Fig. 4b–d), showing a fine modulation within both high (Extended Data Fig. 4c) and low (Extended Data Fig. 4d) nucleosome concentrations, in line with the limited presence of open chromatin regions near NPCs^31^ or within LADs^32^. In wild-type (WT) MEFs, this measurement revealed a specific nucleosome pattern, which showed a gradual increase in nucleosome concentration at a distance of ~20 to ~60 nm from the lamin filaments, followed by a constant nucleosome concentration farther away (Fig. 1c). This suggested a less dense chromatin organization at the NL–chromatin interface, where LADs interact directly with the lamina, than 60–100 nm away from the NL. Interestingly, the concentration of nucleosomes was not linearly correlated with the local concentration of lamin filaments at the NL (Fig. 1d and Extended Data Fig. 4e).

**Table 1:** Cryo-ET data collection and processing

	In situ nucleosome consensus average (EMD-52630)	In situ nucleosome canonical average (EMD-52633)
**Data collection and processing**		
Magnification	×64,000	×64,000
Voltage (kV)	300	300
Electron exposure (e^−^ per Å^2^)	160	160
Electron dose per tilt image (e^−^ per Å^2^)	3.9	3.9
Energy filter slit width (eV)	20	20
Tilt range (°)	−60 to 60	−60 to 60
Tilt increment (°)	3	3
Tilts per tomogram	41	41
Acquisition scheme	Dose-symmetric	Dose-symmetric
Initial subtomograms	130,000	130,000
Final subtomograms	103,173	13,255
Defocus range (μm)	−4	−4
Pixel size (Å)	4.4	2.2
Symmetry imposed	*C*_1_	*C*_1_
Map resolution (Å)	15	13
FSC threshold	0.143	0.143
Map resolution range (Å)	10–20	6–20

Next, we measured the position of individual nucleosomes as a function of their distance from their closest lamin filament in every reconstructed nuclear volume, as shown in Extended Data Fig. 4f. In half of the nuclear volumes, the highest nucleosome concentration was located within a narrow range of 35–47 nm from the NL (Fig. 1e) and remained at similar levels for the next ~50 nm (Extended Data Fig. 4g). We also measured the distance from each lamin filament to its nearest nucleosome (Extended Data Fig. 4h), finding an average minimal lamin–nucleosome distance of 22 ± 5 nm across all tomograms (Fig. 1f and Extended Data Fig. 4i). This analysis further showed that 1.6% of nucleosomes are located within 10 nm of a lamin filament (Extended Data Fig. 4i, shadowed in gray). Representative examples of nucleosomes in direct contact with lamin filaments from this population are shown in Fig. 1g.

A previous study described that chromatin is excluded from the vicinity of NPCs^33^. We investigated the concentration of nucleosomes around NPCs and its correlation with the lamin meshwork density. To this end, we determined the coordinates of NPCs and measured the concentration of nucleosomes as a function of the distance to the center of the closest NPC (Fig. 1h–i). Given that the diameter of NPCs is ~110 nm in MEFs^34^, we measured the local concentration of nucleosomes within a radial distance of 60 to 160 nm from the NPCs’ central coordinates. The nucleosome concentration increased with increasing distance from the NPCs, reaching the previously measured maximal values (Fig. 1c) only at a distance of >150 nm. However, the concentration of lamin filaments was less affected by distance from NPCs (Fig. 1j). Conclusively, lamin filaments are affected by specific NPC components^35^; however, their concentration is steady around NPCs. These results suggested that variations in chromatin density at the nuclear–lamina interface require high-resolution approaches, such as cryo-electron tomography (cryo-ET), to be detected.

### The effect of lamin isoforms on the NE

**Fig. 2:**
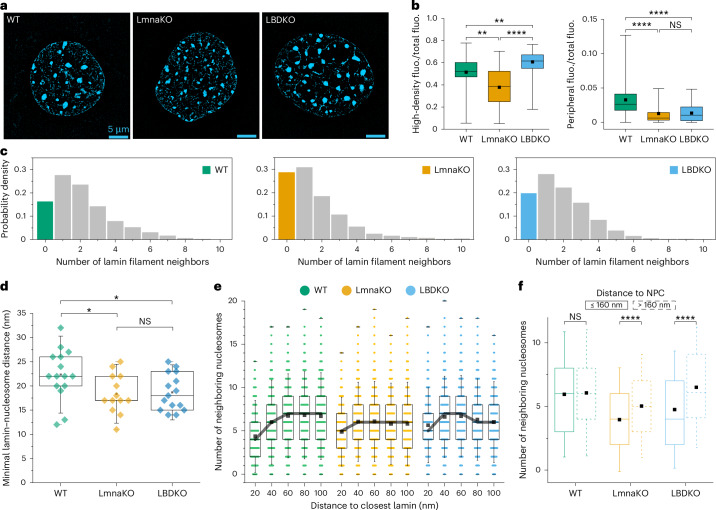
Alterations of nuclei, filaments and nucleosome distribution in lamin-KO cells. **a**, The chromatin in WT, LmnaKO and LBDKO MEF cells was stained by DAPI and imaged by 3D-SIM. **b**, Left: quantification of high-density fluorescent chromatin foci exhibited significantly lower overall fluorescence intensity in LmnaKO nuclei compared to LBDKO and WT nuclei. Right: quantification of DAPI signal revealed a reduction in peripheral chromatin in both lamin-KO cells compared to WT cells. The signal was quantified within the 40% distal area of nuclei. The numbers of nuclei assessed for volume calculation were 48 and 35 for LmnaKO, 89 and 47 for LBDKO and 47 and 36 for WT cells. **c**, The meshwork density of lamin filaments is shown. The number of neighboring filaments detected at <24 nm around each lamin segment is plotted. The fraction of lamins without neighboring filament was significantly increased from 16% in the WT to 28% in LmnaKO cells and 19% in LBDKO cells, with a drop in the mean neighbour count of 2.1, 1.6 and 1.9 in each cell line, respectively (Extended Data Fig. 4j). **d**, The overall minimal lamin-to-nucleosome distances are plotted for each cell line (as in Fig. 1f). Each data point represents the highest nucleosome density distance per tomogram. The medians of the minimal lamin-to-nucleosome distances were 18.4 ± 4.1 nm for LmnaKO cells and 18.7 ± 3.8 nm for LBDKO cells, corresponding to ~82% of the WT’s mean. Each colored diamond indicates the average minimal lamin–nucleosome distance in a single tomogram. **e**, The concentration of nucleosomes as a function of their distance from the NL (as in Fig. 1c). The lamin-independent average nucleosome concentration is presented in Extended Data Fig. 4k. Gray lines connect the median values of each column. **f**, For each cell line, nucleosomes are grouped into two by a distance to the center of the closest NPC of ≤160 nm or >160 nm. All box plots show a box between the 25th and 75th percentiles, the median as a horizontal line, the mean as a black square and 1.5 s.d. as whiskers. Significance was calculated using a one-way ANOVA. ****P < 0.0001, ***P < 0.001, **P < 0.01 and *P < 0.05; NS, *P* > 0.05. For the analysis shown in **c**–**f**, 15 WT, 13 LmnaKO and 15 LBDKO tomograms were used.

**Extended Data Fig. 5:**
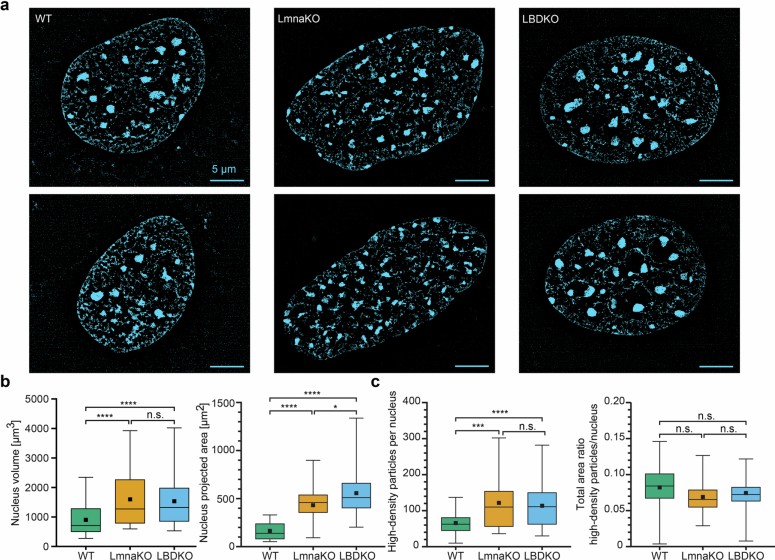
Changes in chromatin architecture in lamin knockout MEF cells. **a**. Super resolution structured illumination microscopy, 3D-SIM, images of WT, LmnaKO, LBDKO MEF cells. Chromatin was stained with DAPI. **b**. Quantification of nuclear volumes indicated increase in both lamin knockout cells (left). Quantification of nuclear area (using projection images) showed a slight increase in the lamin knockout cell lines, compared to the WT (right). **c**. The number of high-density fluorescence chromatin foci, exhibited a significant increase in both LmnaKO and LBDKO compared to WT nuclei (left), while the total area of foci normalized to the nucleus area remained similar across all cell lines (right). The number of nuclei assessed were 35 for LmnaKO, 47 for LBDKO, and 36 for WT cells. Data were from two distinct experiments. All boxplots show a box between the 25th and 75th percentiles, the median as a horizontal line, the mean as a black square, and whiskers represent 1.5 standard deviations. Significance was calculated using a one-way ANOVA. p-value < 0.0001(****), p-value < 0.001(***), p-value < 0.05 (*), and n.s. p-value > 0.05.

To investigate the effect of A-type and B-type lamins on chromatin structure, we firstly applied super-resolution microscopy^36^ to visualize the global chromatin organization in WT, *Lmna*^−/−^ (LmnaKO) and *Lmnb1*^−/−^;*Lmnb2*^−/−^ (LBDKO) MEFs (Fig. 2a and Extended Data Fig. 5a). KO cell lines exhibited slightly enlarged nuclei (Extended Data Fig. 5b) and altered high-density chromatin foci, highlighting the role of lamins in maintaining nuclear architecture (Fig. 2b, left, and Extended Data Fig. 5c). Quantitative analysis of the fluorescence images indicated that peripheral chromatin staining was reduced in both lamin-KO cells (Fig. 2b, right). To further explore the molecular organization of the nuclear periphery at higher resolution, we used cryo-FIB milling in conjunction with cryo-ET and image analysis, as described for WT MEFs. Lamins were segmented from 13 LmnaKO and 15 LBDKO MEF tomograms and the local concentrations of lamin filaments were determined (Fig. 2c and Extended Data Fig. 3b,c). While the lamin meshwork underlying the NE appeared visually similar, substantial differences were observed in the proportion of single lamin filaments without neighboring filaments. In WT MEFs, the majority of lamin filaments were organized into a closely packed meshwork, with only 16% of isolated filaments (Fig. 2c). In the absence of A-type lamins, up to 28% of filaments were isolated, whereas the removal of B-type lamins resulted in a modest increase to 19% of lamins lacking closely neighboring filaments (Fig. 2c). An additional effect of lamin A/C KO was the overall decrease in the mean count of neighboring filaments, from 2.1 in WT to 1.6 in LmnaKO cells (Extended Data Fig. 4j). Interestingly, the lamin meshwork density in LBDKO cells was not substantially altered (Extended Data Fig. 4j), possibly reflecting the enrichment of A-type over B-type lamins in these cells.

The decrease in the minimal distance between lamins and nucleosomes demonstrated that nucleosomes were positioned closer to the NL in nuclei lacking one of the lamin types (Fig. 2d). This may be attributed to the lower occupancy of lamins and their associated factors at the NE in lamin-KO cell lines. Surprisingly, the overall nucleosome concentration was significantly reduced in LmnaKO cells, within 100 nm from the lamina, while remaining unchanged in LBDKO cells (Fig. 2e and Extended Data Fig. 4k). In LmnaKO cells, nucleosome concentrations peaked at a shorter distance of ~40 nm from the NL and did not reach the same concentration level as observed in WT cells (Fig. 2e). Notably, LBDKO cells exhibited a surge in nucleosome concentration at 40–60 nm from the NL, followed by a decline in nucleosome density, reaching concentration levels comparable to those in LmnaKO cells at 80–100 nm. These findings indicated a correlation between A-type lamin and a denser chromatin architecture at the nuclear–lamina interface that may be mediated by a direct molecular lamin–chromatin interactions.

Moreover, in both KO cell lines, we noted a statistically significant alteration in the concentration of nucleosomes around NPCs. Nucleosomes were less concentrated in the immediate vicinity of the NPC (≤160 nm away from the center coordinates of NPCs) compared to WT (Fig. 2f), indicating that alterations in lamins also affect the chromatin organization around NPCs. Thus, in addition to lamins, other NE structures, such as the NPC, impact local chromatin organization.

### Lamin A interacts with H2A–H2B and nucleosomes

**Fig. 3:**
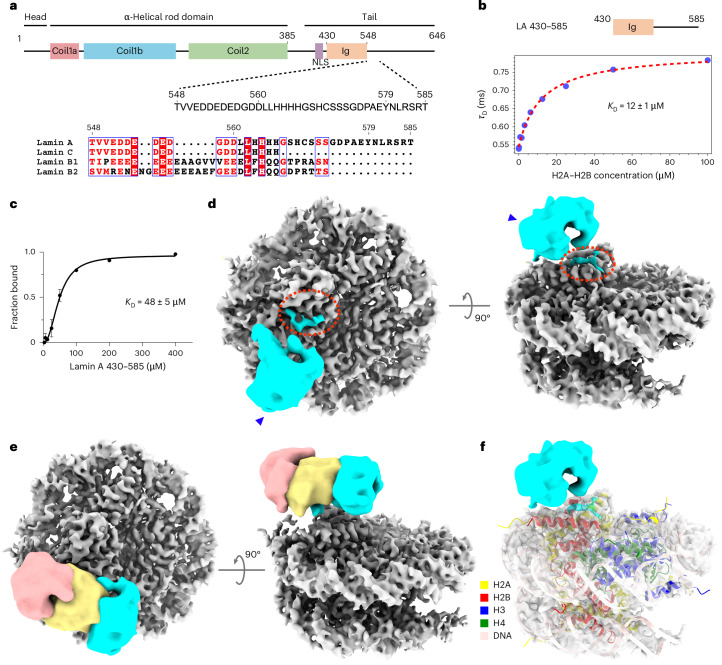
The tail domain of lamin A interacts with H2A–H2B heterodimers and nucleosomes. **a**, A schematic view of the lamin A protein shows the various protein domains and the position of the NLS. The sequence of the lamin A tail domain, amino acids 548–585, is aligned to the other main lamin isoforms: lamin C, lamin B1 and lamin B2. The relatively conserved amino acids are colored. **b**, The affinity of LA 430–585 toward the H2A–H2B heterodimer was determined by FCS (Methods and Extended Data Fig. 6c). **c**, The affinity between LA 430–585 and nucleosomes was quantified by an EMSA (Extended Data Fig. 7b). Data are presented as the mean values ± s.d. **d**, The structure of the LA 430–585 (cyan)–nucleosome (gray) complex was determined by cryo-EM. The densities corresponding to the Ig-like domain (arrowhead) and additional lamin A tail (red dotted oval) were identified. The Ig-like domain was filtered to 6.8 Å. **e**, Variability in the position of the Ig-like domain, filtered to 6.8 Å, was detected across different structural classes (pinkish, yellow and cyan), indicating that the Ig-like domain exhibits greater flexibility compared to the stationary binding site (red dotted oval in **d**). **f**, The nucleosome structure (PDB 6ZHX) fitted into the EM density indicated that LA 430–585 interacts with the H2A–H2B heterodimer. Three independent biological replicates were applied to analyze **b** and **c**. Source data

**Extended Data Fig. 6:**
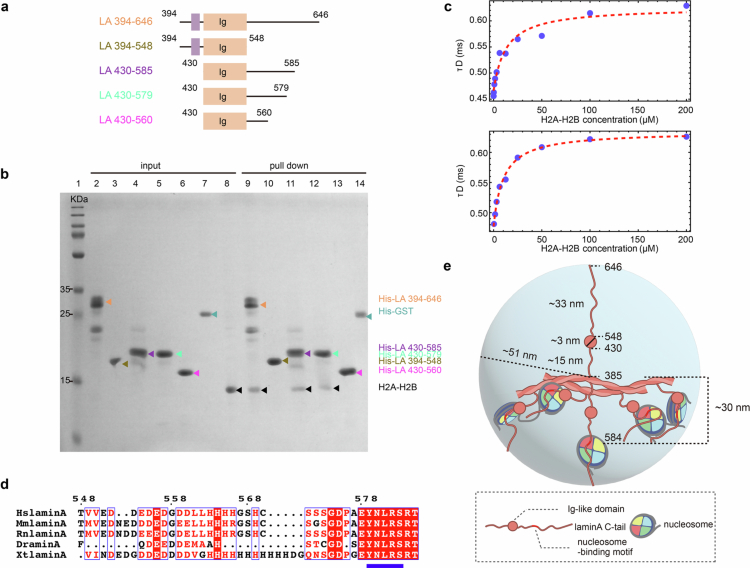
The lamin A tail domain and its interactions with H2A-H2B. **a**. Five truncations of lamin A tail domain were used during in vitro biochemical and structural analyses. **b**. Pull-down assay for identifying the region within lamin A responsible for H2A-H2B binding. 100 ug of each candidate truncation, LA 394-646, LA 394-548, LA 430-585, LA 430-579, LA 430-560 and His-GST as a negative control were immobilized on the 200 µl Ni-NTA resin. 5 µl of each sample was analysed by SDS-PAGE (lane 1-6). Purified H2A-H2B heterodimer was shown in lane 7. 100 µg H2A-H2B was applied to each sample and after several rounds of washing and migrated on the SDS-PAGE (Lane 8-13). **c**. Binding isotherms based on the translational diffusion times observed in FCS measurements as a function of lamin concentration yielded a binding affinity between LA 430-585 and H2A-H2B heterodimer of 12 ± 1 µM, confirmed by three biological replicates. **d**. Sequence alignment of lamin A from amino acid 548 to 585 in 5 different species, HslaminA (homosapiens lamin A), MslaminA (musmusculus lamin A), RnlaminA (Rattusnorvegicus lamin A), DrlaminA (Daniorerio lamin A), XtlaminA (Xenopustropicalis lamin A). It shows that the tail domain of lamin A is highly conserved, especially in the region of 578-585 (underlined in blue). **e**. A schematic illustration shows that the C-terminal laminA domain can be extended to a distance of up to ~51 nm away from the lamin filament. The nucleosome-binding site within the C-terminal laminA domain is located at a distance of ≤30 nm away from the lamin rod domain. Three independent replicates were done for **b** and **c**. Source data

Previous studies showed the possibility of a direct molecular interaction between chromatin and lamins^37,38^ through their tail domains^38–40^. On the basis of sequence alignment (Fig. 3a), we designed a set of lamin A tail truncations (Extended Data Fig. 6a) and studied their binding properties to H2A–H2B heterodimers and reconstituted nucleosomes (Fig. 3b,c). Using pulldown assays (Methods), we found that the C-terminal tail (C-tail) of lamin A (LA 394–646) effectively binds to H2A–H2B heterodimers (Extended Data Fig. 6b). Notably, a truncated tail domain spanning from amino acids 430 to 585 (LA 430–585) exhibited a similar binding property to the full-length tail domain (LA 394–646), indicating that this region is sufficient for binding to H2A–H2B heterodimers. In contrast, truncated tail regions comprising either the nuclear localization sequence (NLS) and the Ig-like domain (LA 394–548) or only the Ig-like domain (LA 430–560) did not bind to H2A–H2B heterodimers, although weak binding was detected with LA 430–579 (Extended Data Fig. 6b). Fluorescence correlation spectroscopy (FCS) measurements indicated a dissociation constant *K*_D_ of 12 ± 1 µM for the binding of LA 430–585 to purified H2A–H2B heterodimers (Fig. 3b and Extended Data Fig. 6c).

**Extended Data Fig. 7:**
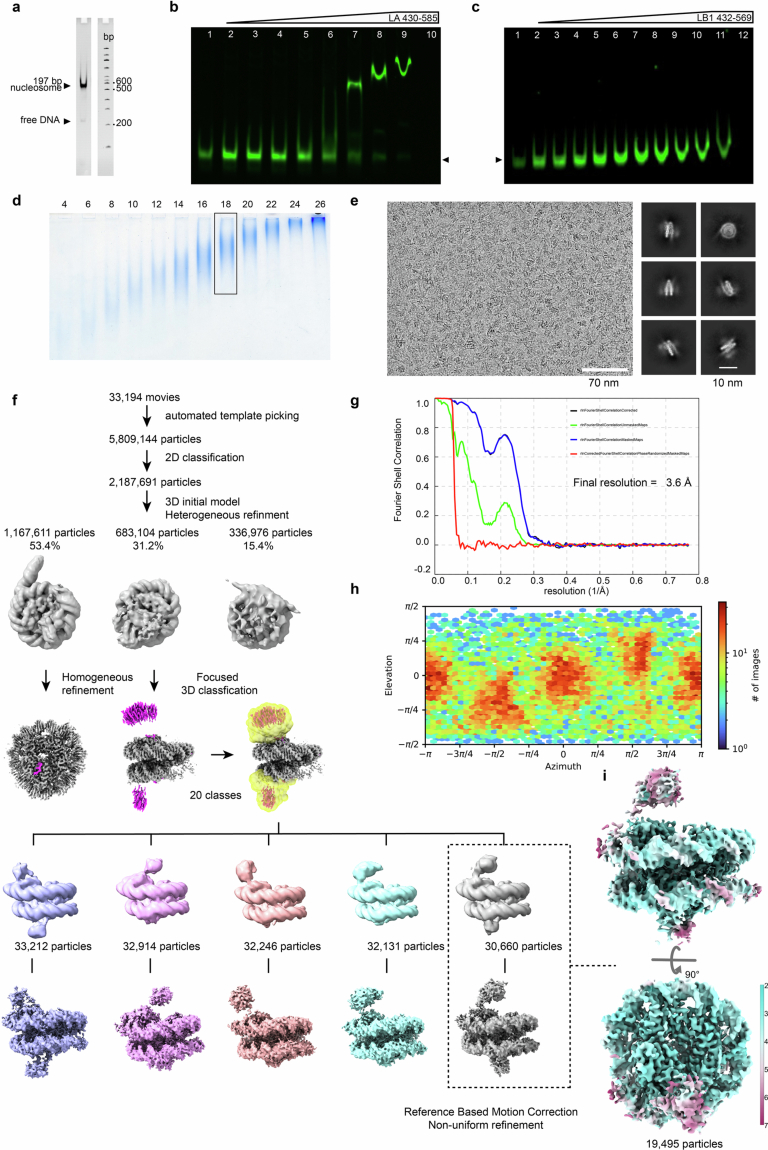
Biochemical and structural analysis of LA 430-585 and nucleosome. **a**. In vitro assembled nucleosome was analysed using a 6% native PAGE. **b**. EMSA of LA 430-585 binding to fluorescently labelled nucleosomes. The nucleosome position was marked by black arrowhead. Data was collected from three biological replicates. This experiment was used to quantify the affinity of interactions in Fig. 3c. **c**. EMSA of LB1 432-569 binding to fluorescently labelled nucleosomes. There is no shifted band, implying no direct interaction between lamin B1 tail domain and nucleosomes. **d**. LA 430-585 and nucleosome complex after Grafix was analysed by SDS-PAGE. Fraction 18 was collected for cryo-EM sample preparation. **e**. Representative micrograph of the dataset used to determine the structure of LA 430-585 and nucleosome complex. (left) 2D class averages generated from the dataset. (right) **f**. Flow chart for image processing by cryoSPARC. **g**. Gold standard Fourier shell correlation (FSC) curves of final 3D reconstitution (3.6 Å). **h**. Angular distribution of particles. **i**. Final reconstruction of the LA 430-585-nucleosome complex coloured by local resolution. Calibration bars are provided. Three independent replicates were done for **b**. Source data

Next, we analyzed the interactions between LA 430–585 and reconstituted nucleosomes^41^ (Extended Data Fig. 7a,b). An electrophoretic mobility shift assay (EMSA) showed direct binding between LA 430–585 and nucleosomes (Extended Data Fig. 7b), while no interactions were detected between nucleosomes and the truncated tail domain of lamin B1 amino acids 432–569 (LB1 432–569) (Extended Data Fig. 7c). We determined a *K*_D_ of 48 ± 5 µM for the binding of LA 430–585 to nucleosomes (Fig. 3c). This modest affinity suggested a dynamic interaction between lamin A and nucleosomes. Subsequently, using GraFix^42^, we purified the complex and conducted cryo-electron microscopy (cryo-EM) analysis. The structure of the LA 430–585–nucleosome complex was resolved to ~3.6-Å resolution using single-particle cryo-EM (Fig. 3d, Extended Data Fig. 7d–I and Table 2). Two separate densities of the lamin A tail domain (Fig. 3d, cyan) were detected in addition to the nucleosome (Fig. 3d, gray). The characteristic density of the Ig-like domain (Fig. 3d, arrowhead) was found in multiple positions in the vicinity of the nucleosome (Fig. 3e and Extended Data Fig. 7f). The other density found attached to the nucleosome (Fig. 3d, red dashed circle) revealed a contact site with the H2A–H2B heterodimer of the nucleosome (Fig. 3f). We identified densities of a tyrosine and an arginine and assigned them to lamin A Y579 and R582. To validate these amino acid assignments, we used a shorter lamin A truncation LA 430–579 and analyzed the structure of the LA 430–579–nucleosome complex by cryo-EM (Supplementary Fig. 2). As expected, the density map showed the canonical nucleosome structure without any additional density, implying that amino acids 579–585 are indispensable for the binding of lamin A to nucleosomes.

**Table 2:** Cryo-EM data collection, refinement and validation statistics

	Nucleosome–lamin A peptide structure (EMD-50114; PDB-9F0O)	Nucleosome–LA 430–585 (EMD-50291)	Nucleosome–LA 430–579
**Data collection and processing**			
Magnification	×130,000	×130,000	×130,000
Voltage (kV)	300	300	300
Electron exposure (e^−^ per Å^2^)	70	70	70
Defocus range (μm)	−0.6 to −2.4	−0.6 to −2.4	−0.6 to −2.4
Pixel size (Å)	0.65	0.65	0.65
Symmetry imposed	*C*_2_	*C*_1_	*C*_1_
Initial particle images (no.)	8,388,318	5,809,144	5,819,637
Final particle images (no.)	588,553	19,495	601,217
Map resolution (Å)	2.3	3.6	2.6
FSC threshold	0.143	0.143	0.143
Map resolution range (Å)	2.3–2.8	3.6–6.7	2.6–3.2
**Refinement**			
Initial model used (PDB code)	6ZHX		
Model resolution (Å)	2.4		
FSC threshold	0.5		
Model resolution range (Å)			
Map sharpening *B* factor (Å^2^)	−81.7		
Model composition			
Nonhydrogen atoms	12,323		
Protein residues	790		
Ligands	0		
*B* factors (Å^2^)			
Protein	72		
DNA	112		
Root-mean-square deviations			
Bond lengths (Å)	0.013		
Bond angles (°)	1.84		
**Validation**			
MolProbity score	0.5		
Clashscore	0		
Poor rotamers (%)	0.46		
Ramachandran plot			
Favored (%)	98.83		
Allowed (%)	1.17		
Disallowed (%)	0		

**Fig. 4:**
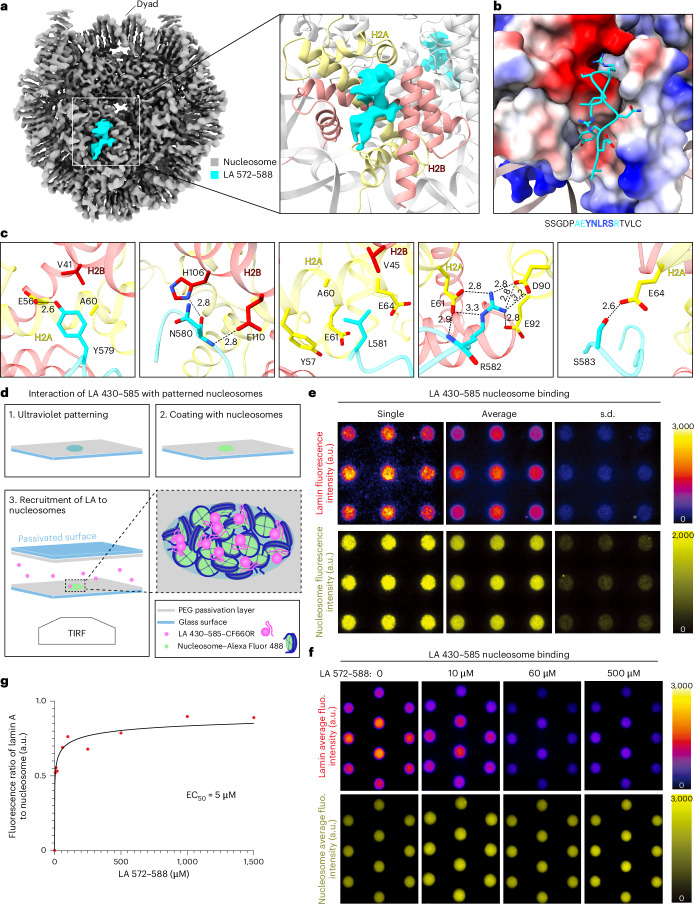
Amino acids 579–583 of lamin A mediate interaction with the nucleosome. **a**, Cryo-EM structural analysis of the peptide LA 572–588 (cyan) interacting with the nucleosome (gray) through the H2A–H2B heterodimer (yellow and pinkish, respectively). **b**, Eight amino acids (AEYNLRSR) of the peptide LA 572–588 were identified in the negatively charged acidic patch of the nucleosome. **c**, Five amino acids (YNLRS) specifically interact with H2A–H2B within nucleosome. This interaction is stabilized by H-bonds, hydrophobic interactions and salt bridges. **d**, A schematic view of the experimental design used for studying lamin A–nucleosome interactions by TIRF microscopy. A passivated glass coverslip was patterned using deep-ultraviolet illumination (1); then, the patterned surface was coated with fluorescently labeled nucleosomes (2). The functionalized coverslip was mounted onto a PEGylated glass slide to fabricate a chamber, where fluorescently labeled LA 430–585 polypeptide was injected at the onset of the binding reaction (3). **e**, The fluorescence intensity of the labeled LA 430–585 polypeptide (top) associated with patterned fluorescently labeled nucleosomes (bottom) for representative single images, averaged images of 14 frames containing a total of 126 patterned spots and related s.d. images. **f**, The LA 572–588 peptide competes with the LA 430–585 polypeptide for nucleosome binding. The unlabeled peptide was added to the reaction mixture described in **d**. The fluorescence intensity of the LA 430–585 polypeptide (top) associated with the patterned fluorescently labeled nucleosomes (bottom) is shown for average images of at least 24 frames. **g**, The fluorescence ratio of nucleosome-associated LA 430–585 against spotted nucleosomes was calculated (Methods), and a curve was fitted with a four-parameter dose–response Hill–Langmuir equation (Methods). The best fit of the overall dataset was obtained for a maximal ratio fixed to less than 1 and showed an EC_50_ of 5 µM. *R*^2^ was 0.78 for the overall dataset. In **e** and **f**, fluorescence calibration bars are provided. All spots are 3 μm in diameter.

**Extended Data Fig. 8:**
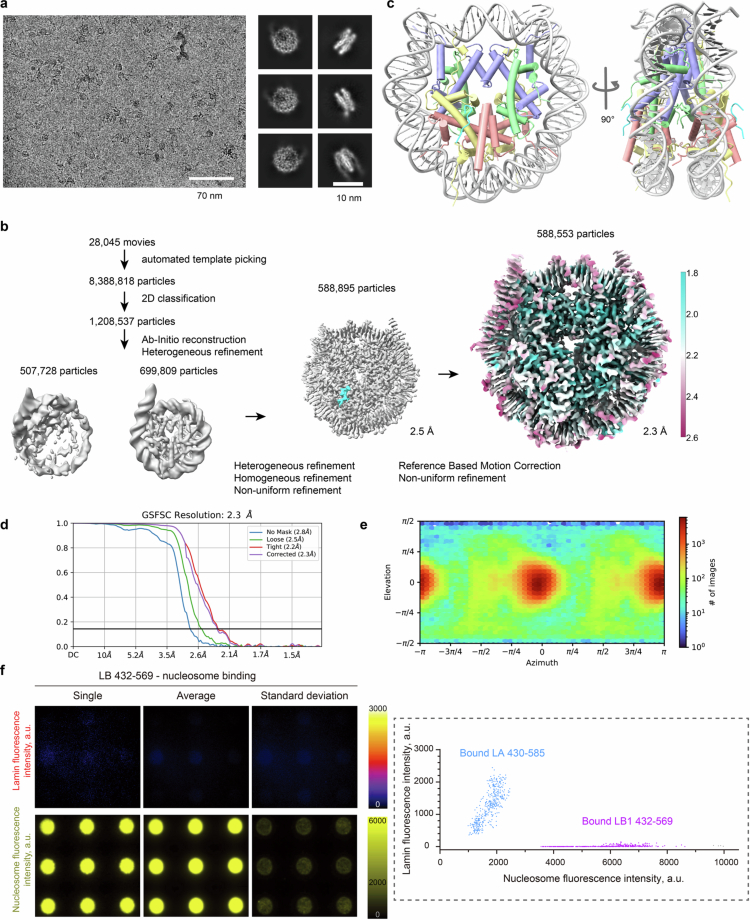
High resolution structural analysis and biochemical binding of peptide (LA 572-588) and nucleosome. **a**. Representative micrograph of the peptide-nucleosome dataset was used to determine the complex structure. Typical 2D class averages of the peptide-nucleosome structure. **b**. Flow chart shows the image processing strategy and final 3D map (2.3 Å) coloured by local resolution. **c**. Gyre and disc views of the peptide-nucleosome structure shows that peptides are localized on the both sides of H2A-H2B positioned in nucleosome. **d**. FSC curve of final 3D map. **e**. Angular distribution of particles. **b**. **e**. Calibration bars are provided. **f**. LB1 432-569 does not bind to nucleosomes. Surfaces functionalized with Alexa fluor 488 labelled nucleosomes were incubated with 1 µM of soluble, CF660R fluorescently labelled LB1 432-569 and imaged after 1 hour. The panels show lamin B1 fluorescence intensity (upper row) associated with the spotted fluorescently labelled nucleosomes (bottom row) for a representative single image, for an average image of 18 frames and a total of 162 spots, and its related significantly low standard deviation image. Fluorescence calibration bars are provided. All spots are 3 μm in diameter. The graph on the right shows that nucleosome-associated lamin fluorescence indicated that lamin B1 (Bound LB1 432-569) fluorescence detected on patterned is negligeable compared to that of lamin A (Bound LA 430-585), even for 4 times more immobilised nucleosomes. For lamin A, 399 fluorescence values ranged between 361 and 2446 a.u., and for lamin B1, 370 fluorescence values ranged between 0 and 142 a.u.

To further explore this interaction, we analyzed the binding of a synthetic peptide comprising amino acids 572–588 of lamin A (LA 572–588) to nucleosomes. Using cryo-EM structural analysis, we resolved the complex at 2.3-Å resolution (Fig. 4a, Extended Data Fig. 8a–e and Table 2). Eight amino acids (AEYNLRSR) of the peptide were resolved at the nucleosome surface (Fig. 4b), while five amino acids (YNLRS) were found to interact with the acidic patch of the H2A–H2B heterodimer (Fig. 4c). The side chain of Y579 forms a hydrogen bond (H-bond) with H2A E56, stabilized by a hydrophobic interaction through H2A A60 and H2B V41. The main chain of N580 forms an H-bond with H2B H106 and E110. The C_βγ_ of H2A E61 and E64, the aromatic ring of H2A Y57 and the side chains of H2A A60 and H2B V41 provide a hydrophobic surface for holding the side chain of L581. Most importantly, R582 acts as an arginine anchor to H2A E61, D90 and E92 through salt bridges. Lastly, the side chain of S583 interacts with H2A E64 through an H-bond. Remarkably, the YNLRS motif is unique to the lamin A tail domain (Fig. 3a) and highly conserved in evolution (Extended Data Fig. 6d). The lamin A tail domain can extend up to ~50 nm from the filament, with the nucleosome-binding site positioned up to ~30 nm away (Extended Data Fig. 6e). Given that our in situ measurements showed an average distance of 22 ± 5 nm between lamin filaments and nucleosomes (Fig. 1f), direct binding between these two components at the nuclear periphery is plausible.

The NL features a high concentration of lamin tail domains along each filament, which can potentially interact with dense nucleosome regions constituting the LADs. To mimic this scenario, we patterned surfaces with fluorescently labeled nucleosomes to create high-concentration spots of nucleosomes. We used a fluorescently labeled LA 430–585 and exposed it to nucleosomes immobilized on patterned surfaces (Fig. 4d). Total internal reflection fluorescence (TIRF) microscopy imaging showed that LA 430–585 bound specifically to the spotted nucleosomes (Fig. 4e), while LB1 432–569 did not, even at higher nucleosome concentrations (Extended Data Fig. 8f). Next, we demonstrated that the presence of the peptide LA 572–588 competed with LA 430–585 for nucleosome binding. This competition was evident from a decrease in fluorescence signals as a function of increasing peptide concentration (Fig. 4f). Quantification of these assays indicated a half-maximal effective concentration (EC_50_) of 5 µM (Fig. 4g). While peptides commonly require higher concentrations for competition, this observation may hint at an additional low affinity of the tail domain to nucleosomes that accounts for the multiple positions of the Ig-like domain found in our structural analysis (Fig. 3e). Overall, these results showed that the direct interaction between lamin A and nucleosome occurs through the tail domain. These interactions are dynamic, as expected for elements undergoing reorganization during the cell cycle.

### A-type and B-type lamins distinctly impact chromatin architecture

**Fig. 5:**
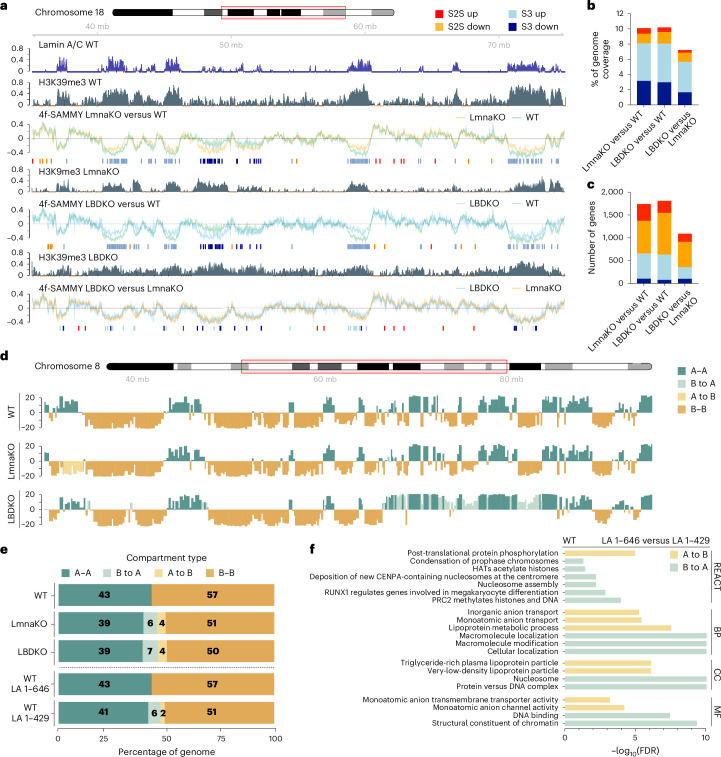
Lamins and C-tail-deficient lamin A induce heterochromatin remodeling. **a**, A representative genomic region of chromosome 18 (40 Mb on chr18: 35,000,000–75,000,000) showing tracks for lamin A/C ChIP-seq, H3K9me3 ChIP-seq and 4f-SAMMY-seq in the WT, LmnaKO and LBDKO cells. 4f-SAMMY-seq solubility profiles comparing the different cell lines are represented as the log of sequencing reads of the more soluble S2S over those of the less soluble S3. The line represents the mean of triplicates and the s.d. is shown as a shadow. Below each track pair, the respective significantly differentially soluble regions are indicated as follows: S2S up, red; S2S down, orange; S3 down, dark blue; S3 up, light blue. **b**, The percentages of the genome affected by the removal of lamin genes are shown as a stacked bar plot, following the color code described in **a**. **c**, Stacked bar plot showing the number of protein-coding genes with altered solubility (Methods). **d**, Chromatin compartment analysis using 4f-SAMMY-seq (Methods). The representative first eigenvector on chromosome 8 (32,000,000–95,000,000) at 50-kb resolution is reported for WT, LmnaKO and LBDKO cells. Regions with concordant (A–A and B–B) or discordant compartment (B–A and A–B) classification in LmnaKO and LBDKO compared to WT are marked. **e**, A stacked bar plot showing average percentages of A and B chromatin compartments in WT. Top, chromatin compartment shifts were detected in LmnaKO and LBDKO cells when compared to WT cells. Bottom, expression of full-length lamin A (WT + LA 1–646) or C-tail-deficient lamin A (WT + LA 1–429) in WT cells, indicating that lamin A tail domain supports B compartment (WT + LA 1–429 compared to WT + LA 1-646 cells). **f**, GO enrichment analysis of genes undergoing compartment changes comparing WT + LA 1–646 to WT + LA 1–429. Bar plots represent significantly enriched Reactome (REACT), biological process (BP), cellular component (CC) and molecular function (MF). HATs, histone acetyltransferases; CENPA, centromere protein A.

**Extended Data Fig. 9:**
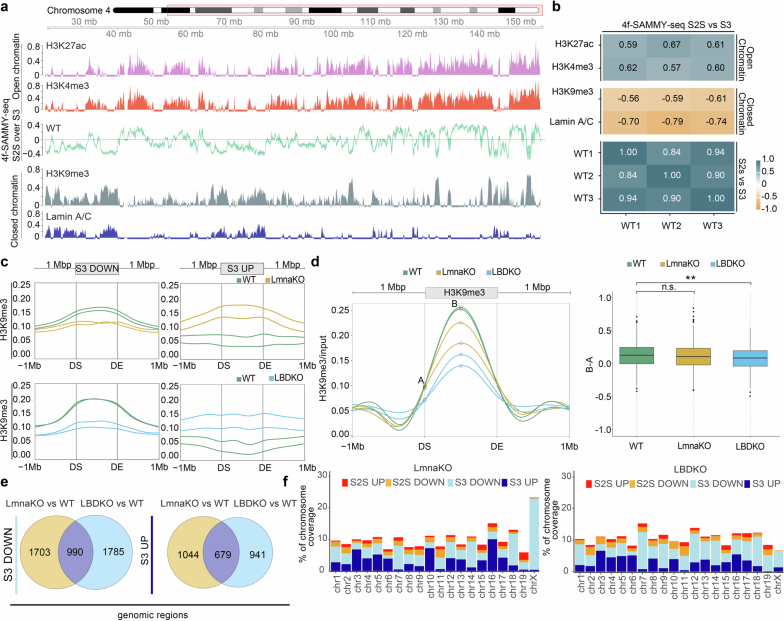
Euchromatin and heterochromatin distribution captured by 4 f -SAMMY-seq. **a**. Distribution along a representative region of chromosome 4 (chr4: 20,000,000-END) of 4f-SAMMY-seq consensus track of WT MEF (S2S vs S3). The continuous line represents the mean across the WT replicates and the lighter colour shadows represents the standard deviation. Positive signal enrichments, defined as the ratio of S2S over S3, are enriched in the most accessible S2S fraction and coincide with euchromatin, while its negative values, enriched in the insoluble S3 fraction, match lamina-associated heterochromatic regions, LADs. The ChIP-seq tracks for histone mark associated to active chromatin (H3K27ac – purple, H3K4me3 - red), constitutive heterochromatin (H3K9me3 –blue) and laminA/C are also shown. For ChIP-seq data, the y axis range is set to zero as minimum value. **b**. Genome-wide Spearman correlation for 4f-SAMMY-seq (S2S vs S3 enrichment) between three WT replicates (WT1, WT2, WT3), histone modifications ChIP-seq (H3K27ac, H3K4me3), laminA/C ChIP-seq with H3K9me3. **c**. Meta-profiles of H3K9me3 enrichment signal over S3 UP and S3 DOWN genomic regions in LmnaKO (top) and LBDKO (bottom). Lines represent two replicates of the H3K9me3 experiment, WT (green), LmnaKO (yellow), and LBDKO (light blue). The x-axis shows the relative position with respect to the start (domain start, DS) and end (domain end, DE) of S3 UP and S3 DOWN genomic regions, along with 1 Mb flanking regions. **d**. Metaprofile of H3K9me3 enrichment within 2 Mb of H3K9me3 domains in WT (green), LmnaKO (yellow), and LBDKO (light blue) MEF cells. The A-B circles illustrate the method used to calculate the relative differences in H3K9me3 ChIP over input enrichment at two points in the H3K9me3 domain: the Domain Start (DS) (point A) and the center of the peak (point B). The box-plot distribution on the right shows the A-B ChIP-seq enrichment for MEF WT (green), LmnaKO (yellow), and LBDKO (light blue) cells. The lower and upper edges of each box correspond to the first and third quartiles, respectively, with the horizontal bar representing the median. Whiskers extend up to 1.5 times the interquartile range (IQR) from the edges. Data points outside this range are outliers and are represented by dots. Statistical analysis was performed using a two-sided Wilcoxon test. WT vs LBDKO p = 0.0062 (**); WT vs LmnaKO p = 0.3757 (n.s.). Two independently processed ChIP experiments were performed for each condition. **e**. Proportional Venn diagram showing the common S3 UP and S3 DOWN genomic regions in LmnaKO and LBDKO. **f**. Distribution of significantly differentially soluble regions across chromosomes in LmnaKO (left) and LBDKO (right) in comparison to WT cells. Colour code: S2S UP (red), S2S DOWN (orange), S3 UP (light blue), S3 DOWN (blue).

To reveal the impact of lamins on genome organization, we examined chromatin structure dynamics using genome-wide sequencing-based techniques, including 4 fractions sequential analysis of macromolecules accessibility sequencing (4f-SAMMY-seq)^24,43^, chromatin immunoprecipitation sequencing (ChIP-seq)^44^ and RNA-seq^45^. Genome-wide correlation analysis between the 4f-SAMMY-seq and ChIP-seq data for open and closed chromatin-associated histone modifications, as well as lamins A/C (Fig. 5a and Extended Data Fig. 9a,b), confirmed that 4f-SAMMY-seq effectively captures lamin-induced changes in chromatin solubility profiles (Fig. 5a and Methods).

**Extended Data Fig. 10:**
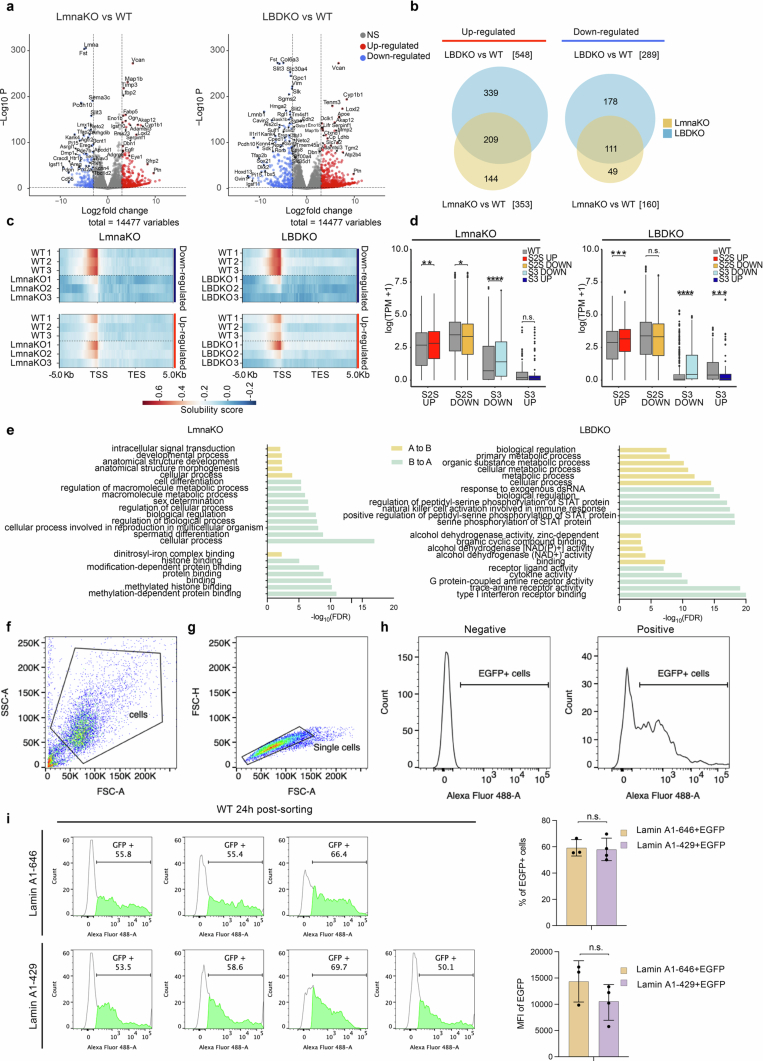
Lamin specific genome alterations. **a**. Volcano plots showing differentially expressed genes (DEGs) between WT and LmnaKO (left), WT and LBDKO (right). The log2FC value > 3, log2FC value < −3 and p-adj < 0.01 (BH) are the cut-off value for significant upregulated (red), and downregulated (blue) Differentially Expressed Genes (DEGs). **b**. Proportional Venn diagram of common up-regulated (left) and down-regulated (right) DEGs in LmnaKO and LBDKO. **c**. Heat maps of solubility profiles of downregulated (above) and upregulated (below) Differentially Expressed Genes (DEGs) aligned to the transcription start and end sites (TSS and TES), shown as triplicates. Red and blue correspond to higher or lower accessibility, respectively. **d**. Box-plot distribution of log2 of transcripts per million (TPM + 1) in significantly differentially soluble regions in LmnaKO (left) and LBDKO (right). The box lower and upper edges are the first and third quartiles, and the horizontal bar is the median. Whiskers extend up to 1.5 times the interquartile range (IQR) from the edges. Data points outside the range are outliers and are represented by dots. Statistical significance was assessed using two-sided Wilcoxon test and labelled as p-value < 0.0001(****), p-value < 0,001(***), p-value < 0.01(**), p-value < 0.05 (*), and n.s. p-value > 0.05. Three independent replicates were performed for each condition. **e**. Gene Ontology enrichment analysis of genes that change compartment exclusively in LmnaKO (left) or LBDKO (right); bar plots represent significantly enriched biological processes. **f**. Exclusion of debris based on FSC-A versus SSC-A. **g**. Exclusion of doublets using FSC-A versus FSC-H. **h**. Gating of EGFP-positive cells using a negative non-transfected negative cells to define the gate. This gating strategy was applied to WT MEF transfected with LA 1-646-EGFP or LA 1-429-EGFP, which were used for 4f-SAMMY-seq assay. Corresponding results are shown in Fig. 5e–f. **i**. Histogram of each replicate of EGFP-transfected cells used for 4f-SAMMY-seq. Bar plots quantifying the percentage of EGFP-positive cells and the mean fluorescence intensity (MFI) in WT cells transfected with LA 1-646-EGFP or LA1-429-EGFP, as measured by flow cytometry. Data are presented as mean with SD. Significance was calculated using a two-tailed unpaired t-test (n.s. p-value > 0.05). Three independent biological replicates were performed for LA 1-646-EGFP and four for LA1-429-EGFP.

Our analysis identified specific LmnaKO and LBDKO genomic regions with significantly altered solubility profiles compared to WT (Fig. 5a–c). Here, positive values of the solubility profile coincide with euchromatin, whereas negative values, enriched in the insoluble S3 fraction, match LADs^43^ (Fig. 5a and Extended Data Fig. 9a,b). KO of both lamins resulted in ~10% genome-wide remodeling with over half of the changes affecting LADs (Fig. 5b). These changes impacted over 1,500 genes (Fig. 5c) and significantly modified gene expression patterns, confirmed by RNA-seq analysis (Extended Data Fig. 10a–d). As expected, a decrease in S3 enrichment coincided with a decrease in H3K9me3-marked heterochromatin (Extended Data Fig. 9c, S3 down). The H3K9me3 signal revealed the different impact of lamin isoforms on constitutive heterochromatin (Extended Data Fig. 9d). Additionally, our analysis indicated that the two lamin-KO cells differed by 7% in chromatin solubility changes (Fig. 5b) but still shared overlapping regions of alteration when compared to the unmodified cells (~36% S3 down and ~ 40% S3 up; Extended Data Fig. 9e). Notably, LmnaKO cells exhibited an extensive loss of heterochromatin specifically on the X chromosome (Extended Data Fig. 9f, S3 down).

On the basis of the biochemical properties of chromatin, we built three-dimensional (3D) compartmentalization maps (Methods), as seen by HiC^46^. Chromatin was segregated into active ‘A’ and inactive ‘B’ compartments for WT and lamin KOs (Fig. 5d). In both KO cell lines, ~10% of the genome underwent compartment changes, characterized by more genome regions shifting from B to A (Fig. 5e). Gene Ontology (GO) analysis revealed that A-type lamins are likely to involve in chromosome condensation, histone binding and modifications, while B-type lamins are associated with cell morphology, homeostasis and transmembrane transport (Extended Data Fig. 10e). These results suggested that A-type and B-type lamins epigenetically regulate lamin-specific domains of the genomic compartments.

To shed light on the importance of lamin A tail domain in chromatin organization, we analyzed genome compartmentalization in MEFs that transiently expressed full-length lamin A (WT + LA 1ç646) or a truncated lamin A lacking the C-tail domain (WT + LA 1–429), both fused to eGFP. We ensured comparable lamin expression levels in both cell lines (Extended Data Fig. 10f–i). Interestingly, compared to WT + LA 1–646, WT + LA 1–429 cells exhibited a 6% shift from B to A and only a 2% shift from A to B, suggesting that the lamin A tail domain supports B compartments (Fig. 5e). Notably, GO analysis revealed that the lamin A tail domain is primarily associated with chromatin structure, nucleosome organization and epigenetic regulation (Fig. 5f). These results were in accordance with our structural analysis.

## Discussion

**Fig. 6:**
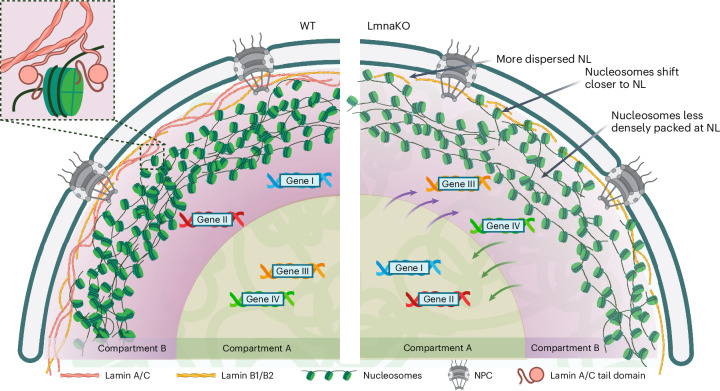
Schematic model of the NE in WT and LmnaKO MEFs. The model shows lamins and chromatin organization underneath the NE in WT and LmnaKO MEF cells. The chromatin (nucleosomes in green) forms a dense structure at the NE by association with the NL, consisting of lamins A/C (red) and lamins B1/B2 (yellow) in WT cells. Top left, lamin A can interact with the nucleosomes through its tail domain. In the absence of A-type lamins (LmnaKO), chromatin remodeling affected the concentration of nucleosomes at the NE, which are less densely packed and shifted closer to the more dispersed NL. These structural effects lead to major nuclear reorganization, including a large number of genes moving from transcriptionally active compartment A to inactive compartment B and vice versa.

The interactions of lamins and chromatin have been studied^14,39,40^; however, the precise structural details of these interactions and 3D imaging of lamin–chromatin interplay at the nuclear periphery remain unresolved. By using state-of-the-art in situ and in vitro approaches, we shed light on how different lamin isoforms modulate nucleosome distribution at the NL and around the nuclear pores, impacting heterochromatin compartmentalization and transcription regulation. As summarized in Fig. 6, our findings suggest that, at the interface between the NL and chromatin, the intrinsically disordered and extensible C-tails of lamin A interact with chromatin through their YNLRS motif (Fig. 6, top left). Although the C-tail domain of lamin A exhibits a modest affinity to nucleosome, the high concentration of lamin A at the lamina, coupled with densely packed nucleosomes in LADs, facilitates continuous, albeit dynamic, lamin√chromatin interactions. In LmnaKO cells, this interaction is tuned down because of the loss of direct or indirect lamin A–chromatin associations. As a result, nucleosome density at the nuclear periphery is reduced (Fig. 6, right), leading to altered heterochromatin organization and compartmentalization.

The expression of lamin A varies across cells and tissues^47^. Therefore, direct lamin A–chromatin interactions are likely to frequently occur in cellular systems with high level of lamin A expression. Additionally, lamin A is also found in the nucleoplasm, where phosphorylated lamins A/C are associated with presumably active transcription chromatin^48,49^. In this case, lamin–nucleosome or lamin–H2A–H2B interactions may take place and function during transcription.

Our structural analysis revealed that the motif YNLRS in lamin A specifically recognizes the acidic patch formed by the H2A–H2B heterodimer within nucleosomes (Fig. 6, top left). The acidic patch is not a known target for histone modifications, which predominantly occur on the histone tails. The lamin A–nucleosome interaction is presumably independent of histone modifications. However, previous studies demonstrated that the depletion of lamin A reduces the activity of methyltransferase Suv39h1, resulting in decreased levels of H3K9me3 (ref. ^50^). Therefore, it is plausible that, upon binding to nucleosomes, the Ig-like domain of lamin A may facilitate the recruitment of methyltransferases, thereby contributing to the maintenance of highly compacted heterochromatin. Importantly, the structural positioning of the Ig-like domain within the lamin A tail presumably enables the recruitment of chromatin-associated factors without interfering with its nucleosome-binding function.

Most importantly, this interaction is unique to lamin A and appears to be evolutionarily conserved, as other lamin isoforms lack the YNLRS sequence motif. Hence, this study suggests a functional distinction between lamin A and other isoforms. Consequently, the lamin A–chromatin interaction leads to reduced dynamics of lamin A compared to lamin C, as shown previously^51^. Indeed, the removal of a fragment of lamin A that contains LA 572–588 increases its mobility at the NL^52^. Moreover, several mutations impacting the YNLRS motif and the surrounding region have been identified in persons with laminopathy, including R582C (ref. ^53^), R582H (ref. ^54^), S583L (ref. ^55^) and R584H (ref. ^56^). These substitutions are associated with familial partial lipodystrophy, while the E578V and C588R (ref. ^57^) substitutions are linked to atypical progeroid syndrome. Together, these disease-linked mutants underscore the importance of the YNLRS motif in lamin A.

The lamin A tail domain is composed of two intrinsically disordered amino acid stretches, 45 and 98 aa in length, separated by a well-structured Ig-like domain (Fig. 3a and Extended Data Fig. 6e). These long, intrinsically disordered regions enable the lamin A tail domain to extend up to ~50 nm. The position of the nucleosome-binding motif in the lamin A tail can be localized up to 30 nm from the NL. This extension would allow the tail domain to be associated with other factors while bound to nucleosomes. For example, the barrier to autointegration factor simultaneously binds to DNA and Ig-like domain, potentially enhancing lamin A binding to nucleosomes^58^. Similarly, intrinsically INM proteins (for example, Emerin, LEMD2 and LBR)^59^ exhibit comparable behavior.

The organization of chromatin at the NE allows cells to regulate gene expression and other essential nuclear functions^17^. Here, we revealed the alteration in spatial nucleosome concentration at the NE as a consequence of the KO of specific types of lamins in MEFs. The surface of the NL is enriched with flexible and unstructured protein domains, primarily the C-tails of lamins and their associated proteins. KO of lamins creates additional vacant space at the NE, allowing nucleosomes to localize in the vicinity of the lamina. In LmnaKO cells, nucleosome concentration is reduced at a distance of 40–60 nm from the lamina. In contrast, loss of B-type lamins leads to an increased nucleosome concentration within this same range, suggesting that A-type lamins have a key role in promoting nucleosome accumulation at the nuclear periphery. In WT cells, chromatin at the NL, primarily consisting of LADs, is enriched with heterochromatin histone methylation marks and only hosts a small fraction of expressed genes^60^. However, our data revealed, even in WT MEFs, local variations in the concentration of nucleosomes at the NE, which may correlate with more open chromatin, typically associated with expressed genes.

Nuclear lamins and chromatin synergistically form a mechanical functional unit to overcome cellular and environmental stress^61^. This synergy relies on tight interactions between these two structures. Cells that express different isoforms of lamins show variations in the lamin meshwork. Notably, we found a reduced concentration of nucleosomes (that is, chromatin) at the NL of MEF cells that express solely B-type lamins. This coincides with previous observations indicating that A-type lamins are mostly expressed in differentiated cells, where the interactions of tightly packed heterochromatin with the NL are enhanced^62^. Consistently, only modest changes in nucleosome concentration were detected upon the removal of B-type lamins, suggesting that A-type lamins exert a prominent influence on chromatin organization at the NE (Fig. 6).

Genome-wide chromatin analysis revealed that A-type and B-type lamins have a selective role in the functional organization of the genome, predominantly by controlling heterochromatin organization (Fig. 5). In agreement with structured illumination microscopy (SIM) and cryo-ET analyses, the absence of A-type lamin results in rearranged global chromatin organization, including changes in LADs, and a reduction in H3K9me3 levels^63^. These alterations correlate with our genomic analyses showing that A-type lamins influence heterochromatin solubility and impair transcription from these regions. Interestingly, the absence of B-type lamins leads to a loss in H3K9me3 domain boundaries. Overall, A-type and B-type lamins display distinct patterns of transcriptional regulation, suggesting their involvement in specific epigenetic pathways and underlying the importance of native lamin expression in retaining functional nuclei. Most importantly, the expression of C-tail-deficient lamin A, lacking the YNLRS lamin A nucleosome-binding motif, reducing densely packed chromatin B compartments. This may explain the reduced nucleosome concentrations observed at the nuclear periphery of LmnaKO cells. Moreover, it suggests a substantial alteration of chromosome organization in agreement with single-cell fluorescence analysis in MEFs^64^ and previous genome-wide analysis^65^.

In summary, we used in vitro and in situ structural analysis to reveal the molecular basis of lamin–chromatin interactions in conjunction with genome-wide analysis. Our findings demonstrate the lamin-dependent organization of the 3D genome and its impact on gene expression variability. A-type and B-type lamins exhibit differential regulatory roles in chromatin architecture, with lamin A distinguished by its direct interaction capabilities with nucleosomes. Therefore, retaining the native levels of each lamin isoform is important for the optimal function of cells.

## Methods

### Statistics and reproducibility

No statistical methods were used to predetermine sample size. No data were excluded from the analyses. The data met the assumptions of the statistical tests used; normality and equal variances were formally tested. The experiments were not randomized, and investigators were not blinded to allocation during experiments or outcome assessment.

### Cells and cell culture

WT, Lmna^−/−^, and Lmnb1^−/−^+Lmnb2^−/−^ MEFs were grown at 37 °C in a humidified incubator with 5% CO2 in high glucose DMEM (Sigma-Aldrich, D5671) supplemented with 10% FCS (v/v), 2 mM L-glutamine (Sigma-Aldrich, G7513) and 1% penicillin-streptomycin (Sigma-Aldrich, P0781). Cells were seeded onto glow discharged cryo-EM grids (Quantifoil 2/1, 200 mesh, Au) overnight. Grids were washed in 1xPBS (Fisher bioreagents, BP399-1) before manual plunge freezing into liquid ethane cooled by liquid nitrogen. Grids were stored in liquid nitrogen before further use. WT, Lmna^−/−^, and Lmnb1^−/−^+Lmnb2^−/−^ cells were cultured in parallel, with each cells consisting of >3 independent biological replicates.

### Cryo-FIB milling

Grids were clipped into CryoFIB AutoGrids (Thermo Fischer Scientific, USA) before being transferred to the FIB-SEM loading station. The grids were mounted onto a custom-built grid holder before being sputter coated with 8 nm Pt/C in a Leica BAF060 system cooled to −160 °C and directly inserted into the Zeiss-Auriga 40 Crossbeam FIB-SEM with a stage temperature of −150 °C. Before milling, grids were covered with 2×5 s flashes of organometallic platinum with the internal gas injection system. Milling was performed at a stage angle of 18 ° and a FIB beam current from 240 to 20 pA at a constant voltage of 30 kV. The process was controlled with the Nano Patterning and Visualization Engine software (Zeiss) and progress was observed with the SEM. Grids with finished lamellae with a thickness of 100–200 nm were stored in liquid nitrogen until further usage.

### Cryo-ET

Grids with FIB-milled lamellae were transferred to a Titan Krios EM instrument (Thermo Fischer Scientific, USA) equipped with a Gatan K2 summit direct electron detector and a Gatan energy filter operated at 300 kV in zero-loss mode. Tomograms of the NE were acquired with a magnification of 64000x (0.21 nm pixel size) with −4 µm defocus and a dose symmetric tilt scheme^66^ from ±60 ° in 3 ° increments and a total dose of 160 e^−^/Å^2^ in SerialEM^67^.

Drift-corrected tilt series were aligned and CTF corrected with IMOD^68^ using either platinum depositions as fiducials or the IMOD patch tracking for alignment. For subsequent lamin and nucleosome detection, tomograms were reconstructed with WBP in bin 4 with a SIRT-like filter applied.

### Cryo-ET image processing and data analysis

Total 100 WT, 53 LmnaKO, 52 LBDKO tomograms from 90 cells were collected. The final analysis was conducted on 43 tomograms with the highest signal to noise ratio, because of the similar thickness of the lamellae (~150 nm). Nucleosomes were detected using a crYOLO^69^ model trained on a data subset of 10 tomograms and applied on the full dataset from all three cell lines. This resulted in 130000 initial nucleosome coordinates, which were imported into MATLAB R2019b (MathWorks, USA) using the TOM toolbox^70^ where subtomograms of 16x16x8 pixels were projected in the z-direction to generate 2D particles^71^. Subsequently, with these particles a 2D classification was performed with RELION 4.0.1^34,72^ to remove obvious non-nucleosome particles and particles that could not form reasonable 2D classes(Supplementary Fig. 1a).

In order to estimate the ratio of false-positive nucleosomes in the remaining subtomograms after 2D classification (103173 particles), we defined the cytoplasmic (false-positive) picking ratio (CPR) as the ratio between the number of false-positive particle coordinates in the cytoplasm and the total number of picked particles. To measure the CPR (Extended Data Fig. 1), we overlaid the particle coordinates with the tomographic data and annotated the particle coordinates, which were localized in cytoplasmic regions of the tomograms. This procedure was implemented as a graphical user interface in MATLAB R2019b.

For the calculation of the in-situ nucleosome consensus average (Extended Data Fig. 2b) the subtomograms were reconstructed with a binning factor of 2 (voxel size 4.4 Å) and a box size of 64x64x64 voxels. The subtomograms were processed using a Refine3D job in RELION 5.0^73^ with 7.5° initial angular sampling, an initial offset range of 5 pixels, and the maxsig parameter set to 300. The initial reference for 3D refinement was created by low-pass filtering the nucleosome structure EMD-33132 ref. ^74^ to 40 Å (Extended Data Fig. 2a). During the alignment process, a reference mask to minimize the influence of neighbouring nucleosomes was applied. This mask was created with the RELION command *relion_mask_create* based on the initial reference by extending the binarized reference of 4 voxels and adding a soft edge of 8 voxels. The resolution of the in-situ nucleosome consensus average (Extended Data Fig. 2b) reached 15 Å (Supplementary Fig. 1b, d), which was estimated based on gold-standard Fourier shell correlation (FSC) using the RELION command *relion_postprocess*.

For 3D classification (Extended Data Fig. 2c) the subtomograms were processed with a Class3D job in RELION 5.0. In this calculation the number of classes was set to 40, the regularization parameter to 1, the angular sampling interval to 7.5°, the offset search range to 5 pixels, and the maxsig parameter to 300. The same initial reference and reference mask were used as described above. The 3D classification was stopped after 17 iterations. The class which attracted the most subtomograms (13255 particles) was selected for further processing.

Subsequently, the selected particles were processed with an identical Refine3D job as described above for the consensus average (except the maxsig parameter was increased to 2000), starting again with the 40 Å low-pass filtered initial reference. Next, the selected particles were reconstructed without binning (voxel size 2.2 Å) and a box size of 128x128x128 voxels. In order to calculate the final in-situ nucleosome canonical average (Extended Data Fig. 2d) the unbinned subtomograms were processed with another Refine3D job in RELION 5.0. Here an initial angular sampling of 7.5°, an initial offset range of 5 pixels, and a maxsig parameter of 2000 were set. As reference map the result of the preceding 3D refinement was used, rescaled to a voxel size of 2.2 Å and low-pass filtered to 18 Å. This structure was also used to create the reference mask for this Refine3D job with *relion_mask_create* by extending the binarized reference of 4 voxels and adding a soft edge of 8 voxels. The resolution of the final in-situ nucleosome canonical average (Extended Data Fig. 2d) reached 13 Å (Supplementary Fig. 1c, e), which was estimated based on gold-standard FSC using *relion_postprocess*.

Lamins were manually segmented using IMOD’s^68^ 3dmod software. To be able to properly follow the filaments, the tomograms were rotated using the slicer viewer to bring the lamina into the viewing plane. Resulting filament coordinates were imported into MATLAB R2019b (MathWorks, USA) and resampled into equidistant coordinates with a 3.5 nm distance using the interparc function^75^. This resulted in 44880 lamin coordinates across the three cell lines. Additionally, in the tomograms containing one or more NPCs, the centres of the NPCs were defined as coordinates for further measurements.

All distance and neighbourhood measurements were performed in MATLAB R2019b (MathWorks, USA). For all measurements, we excluded nucleosomes that were further away from the NL than 100 nm because of the different field of views between tomograms and to focus on the lamin-peripheral nucleosomes. All neighbourhood measurements were done by measuring the number of neighbours in a sphere with a 24 nm radius, a volume that host twice the maximum nucleosome diameter (12 nm) from each measure coordinates.

Graphs were drawn with Origin 2018 (OriginLab, USA) and 3D visualization was performed with ChimeraX 1.7^76^. Additionally, the Artiax^77^ plugin was used for the NPCs.

Histone protein expression, purification and heterodimer assembly.

Xenopus laevis histones, H2A, H2B, H3, and H4, were expressed and purified as previously described^41^. H2A and H2B or H3 and H4 were mixed separately at equal molar ratios, followed by dialysis using refolding buffer (2 M NaCl, 20 mM Tris-HCl pH 8.0, 1 mM DTT). Insoluble components were pelleted by high-speed centrifugation. Soluble H2A–H2B heterodimers and H3–H4 tetramers were purified using size exclusion chromatography (SEC).

### DNA preparation, nucleosome assembly and purification

The 197 bp DNA fragment was designed based on the 147 bp 601 wisdom nucleosomal DNA^78^ and cloned into the PUC57 plasmid. Large-scale PCR amplification was performed, followed by purification using the phenol-chloroform DNA extraction method. DNA was then precipitated in 70% ethanol and resuspended in 20 mM Tris-HCl pH 8.0, 0.5 mM EDTA buffer (TE buffer) for subsequent nucleosome reconstitution.

*Xenopus laevis* histones were assembled into nucleosomes as previously described^41^. Following the removal of dissolved components by centrifugation, the supernatant was concentrated and subjected to further purification using a Superdex™ 200 Increase 10/300 GL column (cytiva). The fractions obtained were analyzed by SDS-PAGE.

### Fluorescence labeling of nucleosome with Alexa Fluor™ 488

Nucleosomes containing a cysteine substitution at H4K20 (H4K20C) were reconstituted and purified using established protocols. Then the H4K20C nucleosome was dialyzed into PBS buffer, followed by two rounds of dialysis. The labeling process involved incubating the H4K20C nucleosomes with Alexa Fluor™ 488 C_5_ Maleimide (Thermo Fisher Scientific, A10254) dye at a 5:1 molar ratio overnight at 4 °C. To quench the labeling reaction, DTT was added to the sample at a final concentration of 10 mM. Finally, free dye was separated from the labeled nucleosomes using glycerol gradient ultracentrifugation, using a 10% to 30% glycerol gradient.

### Constructs of lamin tail domains and protein purification

DNA sequences encoding for lamin A 394–646 (LA 394–646), 394–548 (LA 394–548), 430–585 (LA 430–585), 430–579 (LA 430–578), 430–560 (LA 430–560), laminB1 432–569 (LB1 432–569) were cloned into pET28 vector. Additionally, DNA sequences for LA 430–585 and LA 430–579, intended for structural analysis, were inserted into the 6xHis-SUMO plasmid. For FCS experiments and TIRF microscopy, the LA 430–585 C570A mutation was prepared in the 6xHis-SUMO plasmid to allow specific labeling of CF660R SE dye at position C522. Lamin B1 432–569 (LB1 432–569) was also cloned into the same plasmid.

The above constructs were transformed into the BL21(DE3) bacterial strain and cultured in TB medium at 37 °C. Protein expression was induced by adding 0.5 mM IPTG, followed by 16 h culturing at 18 °C. Harvested bacteria were lysed into 500 mM NaCl, 20 mM Tris-HCl pH 8.0, 25 mM imidazole buffer. Bacteria were lysed by sonication followed by centrifugation at 20,000 g for 1 h. The supernatants were applied to Ni-NTA affinity chromatography. Targeted proteins were eluted by a linear gradient with elution buffer (500 mM NaCl, 20 mM Tris-HCl pH 8.0, 500 mM imidazole). The His-SUMO-tagged proteins were subjected to ULP1 protease incubation, followed by dialysis into the imidazole free lysis buffer for 3 h. Next, the samples were purified using Ni-NTA affinity chromatography, allowing the target proteins to be collected and concentrated to the desired concentration. Finally, proteins were further purified by size exclusion chromatography using a HiLoad™ 16/600 Superdex™ 75 pg (cytiva)., The buffer contained 150 mM NaCl, 20 mM HEPES pH 7.5, 2 mM DTT. The samples were accessed by SDS-PAGE, concentrated, and stored at −80 °C.

### Histone H2A–H2B–lamin A tail binding assay

Purified LA 394–646, LA 394–548, LA 430–585, LA 430–579, LA 430–560, along with a His-GST (negative control), were immobilized on the Ni-NTA resin using a buffer containing 150 mM NaCl, 20 mM HEPES pH 7.5, 25 mM imidazole, 0.01% tween. Rigorous washing steps were performed prior to the introduction of equimolar amounts of the H2A–H2B heterodimer to each lamin truncation. The samples were incubated for 1 h followed by extensive washing steps. Subsequently, the resin was boiled and analyzed by SDS-PAGE. Equal protein amounts were used for each truncation to ensure an accurate comparison.

### Glass surface passivation and deep-ultraviolet-mediated micropatterning

Prior to passivation, slides and coverslips (CVs) were drastically cleansed by successive chemical treatments: 2 h in 2% Hellmanex III; rinsing in ultrapure water; 30 min in acetone; 30 min in 96% ethanol; rinsing in ultrapure water. Slides and CVs were dried using a filtered nitrogen gas flow and oxidized with oxygen plasma (3 min, 30 Watt, Femto low-pressure plasma system Type A, Diener electronic GmbH, Germany), just before an overnight incubation in a solution containing tri-ethoxy-silane-PEG (5 kDa, PLS-2011, Creative PEGWorks, USA) 1 mg/ml in 96% ethanol and 0.02% of HCl. mPEG-silane passivated slides and CVs were stored in a clean container and used within a week.

To direct the binding of nucleosomes to predefined positions on glass CVs, we used the micropatterning strategy^79^ by printing adhesive patterns on a protein-repellent surface. mPEG-silane passivated CVs were exposed to short-wavelength ultraviolet radiation (184.9 nm and 253.7 nm, Jelight, USA) for 2 min through 24 × 24 transparent micropatterns printed on a photomask (Compugraphics, Germany), and immediately mounted onto a PEGylated slide using a double-sided tape (3 M electronics). Then, immediately, the fabricated flow chamber was incubated for 5 to 10 min with 0.2 µM of 25% Alexa Fluor 488 labeled nucleosomes, then saturated with 0.8% BSA in a wash buffer containing 20 mM HEPES pH 7.5 and 100 mM NaCl and finally washed in the wash buffer supplemented with 0.08% BSA. Likewise, free nucleosomes were washed away, and any uncoated position was likely saturated with BSA.

### TIRF microscopy imaging

Reconstitution assays were performed using freshly functionalized chambers and the Lamin A/nucleosome reaction buffer, containing 20 mM HEPES pH 7.5, 100 mM NaCl, 0.3% BSA, 0.03% TWEEN 20, 60 mM β-mercaptoethanol, 0.6% methylcellulose, and lamin polypeptides. The final CF660R fluorescently labeled LA 430 – 585 truncation polypeptide concentrations were 1 µM (Fig. 4e). Alternatively, nucleosome-coated patterns were also tested for their binding capacity to the CF660R fluorescently labeled LB1 432 – 569 truncation polypeptides at a final concentration of 1 µM (Extended Data Fig. 8f). The reaction medium was also supplemented with a variable concentration of unlabeled lamin A-derived peptide 572 – 588. This reaction medium was injected into a passivated flow cell at the onset of the reaction, and imaging was performed after 1 h, at steady state. Specifically for Fig. 4, the amount of nucleosome-associated lamin A truncation polypeptide 430 – 585 was calculated from 442, 815, 560, 369, 731, 653, 675, 665, 616, 298 spots for 0, 5, 10, 20, 60, 100, 250, 500, 1000, 1500 µM peptide 572 – 588, respectively, with a SEM of 0.007, 0.003, 0.010, 0.006, 0.002, 0.003, 0.002, 0.002, 0.002, and 0.004, respectively. TIRF images were acquired using a Widefield/TIRF – Leica SR GSD 3D microscope, consisting of an inverted widefield microscope (Leica DMI6000B / AM TIRF MC) equipped with a 160x objective (HCX PL APO for GSD/TIRF, NA 1.43), a Leica SuMo Stage, a PIFOC piezo nanofocusing system (Physik Instrumente, Germany) to minimize the drift for an accurate imaging, and combined with an Andor iXon Ultra 897 EMCCD camera (Andor, Oxford Instruments). Fluorescent proteins were excited using 3 solid-state diode lasers, 488 nm (300 mW), 532 nm (500 mW), 642 nm (500 mW). Laser power was set to 5% for Alexa-labeled proteins, and dyes were excited for 50 ms. Image acquisition was performed with 25 degrees-equilibrated samples and microscope stage. The microscope and devices were driven by Leica LAS X software (Leica Microsystems, GmbH, Germany).

### Image processing and data analysis of fluorescence images

Steady-state images taken after 1 h reaction was processed with Fiji software (NIH). 46 to 88 or 54 steady-state images were taken for LA 430–585 polypeptide +/- LA 572–588 peptide or LB1 432–569 polypeptide, respectively. To determine the ratio of nucleosome-bound lamin polypeptide to the total patterned nucleosomes, macros written in Fiji allowed to first subtract the background for each individual image and quantify the mean fluorescence intensity of lamin polypeptides and related nucleosomes immobilized on patterned spots in each individual image. The fluorescence ratio was then calculated, and data (Fig. 4f) was fitted with a dose–response curve using GraphPad Prism 10 (‘[Agonist] vs. response, Variable slope’). The equation was

$$x=b+\frac{(a-b)\times {x}^{\rm{HillSlope}}}{{{\rm{EC}}_{50}}^{{\rm{HillSlope}}}+{{x}}^{{\rm{HillSlope}}}}$$

where *a* and *b* are plateaus in the units of the *y*-axis, HillSlope describes the steepness of the curve, and EC_50_ is the concentration that gives a response halfway between *a* and *b*. Three parameters, EC_50_, HillSlope, and *b*, were unconstrained. The best-fit values for the three parameters, plateaus, HillSlope, and EC_50_, were calculated from the overall dataset, with the value of the saturation plateau set to less than 1.

### Super-resolution SIM and spinning disk confocal microscopy

To assess nucleus volume and chromatin organization at the microscopic level, overnight cultured cells were first washed in 37 °C-warmed phosphate saline solution (PBS) before being fixed in 3.7% paraformaldehyde (PFA). After removal of the fixative, nuclei were stained with 10 µg ml^−1^ DAPI solution (DAPI, FluoroPure™–grade, Thermo Fisher) and the actin cytoskeleton with one unit of Alexa 568-labeled phalloidin (Thermo Fisher). For observation, labeled fixed cells were embedded in ProLong Glass antifade (Thermo Fisher). Cells with a typical, well-organized actin cytoskeleton, observed in spread MEFs, were used to assess chromatin organization. For SIM imaging, we used an inverted widefield Zeiss microscope (Axio Observer 7 SR RP Stativ for ELYRA 7) equipped with a 63x ZEISS Plan-Apochromat objective (1.4 NA), providing a field of view of 1280 ×1280 pixels (or 82 µm), along with two pco.edge 4.2 sCMOS, a Piezo stage, an Apotome for grid-based optical light sectioning. In combination with the SIM^2^ processing module, Apotome imaging achieves lateral and axial resolution down to ~110 nm and ~300 nm, respectively. For the spinning disk confocal microscopy, we used an inverted widefield Olympus IXplore SpinSR10 microscope, equipped with a 60x UPLSAPO UPlan S Apo objective (1.3 NA), along with two sCMOS cameras, a YOKOGAWA CSU-W1 spinning disk, and an IX3-ZDC2 Olympus Z-drift compensator.

### Image processing and data analysis of SIM and confocal images

The 3D SIM images were reconstituted from z-stacks consisting of 13 phase images for each z-plane with the same method using SIM^2^ processing algorithm (Zeis). The full z range was analyzed with a sharpness setting of 3, Wiener filter and 20 iterations. The high-density fluorescence chromatin particles were segmented using the Trainable Weka Segmentation plugin (Fiji/ImageJ2), and their number, size, and fluorescence intensity, along with the overall size and fluorescence intensity of the nucleus, were measured. The images reconstructed from SIM2 algorithms using the same settings for all samples to avoid any bias into fluorescence intensity comparison, and all reconstruction parameters (number of phases and grid spacing) were consistent across all images. To compare chromatin fluorescence intensity quantitatively, the images were all acquired with the same illumination settings, and for each nucleus, chromatin fluorescence was normalized to the overall nuclear fluorescence intensity. Calculations and figures were performed using MATLAB R2019b and GraphPad Prism 10.

The nucleus volume was calculated from the 3D confocal z-stacks using Imaris software (Imaris 10.2.0 Bitplane). All nuclei were segmented using the same segmentation method obtained after training in Imaris.

### FCS

The CF660R SE (Sigma-Aldrich, SCJ4600053) labeled LA 430–585 C570A and LB1 432–569 were subjected to SEC to remove free dye. FCS measurements were conducted at protein concentrations of 500 pM in a buffer composed of 20 mM HEPES pH 8 and 150 mM NaCl, supplemented with 0.01% Tween20 (to reduce surface adhesion) and 2 mM of both ascorbic acid and methyl viologen as photoprotectants. The samples were measured in µ-Slide chambers (ibidi) on a custom-built confocal single-molecule fluorescence instrument equipped with a UplanApo 60x/1.20 W objective (Olympus) and a red 640-nm diode laser (LDH-D-C-640, PicoQuant) used in continuous-wave mode at a power of 50 µW. Fluorescence photons were separated from scattered light by a triple-band mirror (zt405/530/630rpc, Chroma) and then passed through a 100-μm pinhole. CF660R photons were selected by a dichroic mirror (T635LPXR, Chroma) and an LP647RU long-pass filter (Chroma) and detected by a SPCM-AQRH-14 single-photon avalanche diode detector (Excelitas).

Data analysis was performed using Mathematica (Wolfram Research) with the custom add-on Fretica (available at https://schuler.bioc.uzh.ch/programs/). Correlation curves, *G*(*τ*), were fitted with a model that includes an amplitude, 1/*N*, and a diffusion time, *τ*_D_, for each measurement, and triplet dynamics (with a triplet amplitude, *c*_T_, and a triplet time, *τ*_T_) that is common for all points of a titration:

$$G\left(\tau \right)=1+\frac{1}{N}\frac{1+{c}_{\rm{T}}\exp \left(-\frac{\tau }{{\tau }_{\rm{T}}}\right)}{\left(1+\frac{\tau }{{\tau }_{\rm{D}}}\right)\sqrt{1+\frac{\tau }{{{S}^{2}\tau }_{\rm{D}}}}},$$

where *s* = 6 is the ratio of the axial to lateral radii of the confocal observation volume. The extracted diffusion times, *τ*_D_, were plotted against H2A–H2B concentration, and this curve was fitted with a one-to-one binding model between LA 430–585 C570A and the H2A–H2B dimer. The fit parameters were the equilibrium dissociation constant *K*_D_, the diffusion time of unbound LA 430–585 C570A and the diffusion time of H2A–H2B-bound LA 430–585 C570A.

### EMSA of nucleosome-lamin complexes

EMSA was conducted at 4 °C in a buffer containing 150 mM NaCl, 20 mM HEPES pH 7.5, 1 mM DTT. Alexa 488-labeled nucleosomes were used to assess binding to LA 430–585 and LB1 432–569. A concentration of 0.05 µM of labeled nucleosomes was incubated with progressively doubling amounts of either LA 430–585 or LB1 432–569. The concentration range of LA 430–585 or LB1 432–569 spanned from 3.1 µM to 400 µM. Following 1 h incubation, the samples were loaded onto a 6% native PAGE gel and imaged for 488 nm fluorescence using Fusion Fx Spectra (Vilber). The decreased quantity of nucleosomes was calculated by ImageJ and the curves were fitted by GraphPad Prism 10.

### Cryo-EM grid preparation and data collection

An excess of LA 430–585 was mixed with purified nucleosomes and incubated on ice for 1 h. Grafix method^42^ was applied to prevent aggregation and stabilize the lamin-nucleosome complex. The glycerol gradient ranged from 10% to 30% while the gradient of glutaraldehyde ranged from 0 to 1% in a buffer containing 100 mM NaCl 20 mM, 20 mM HEPES pH 7.5. A total of 200 µl of mixed the complex was loaded and centrifuged at 215,600 g for 16 h at 4 °C. The gradient was manually fractionated using 200 µl per fraction and Tris-HCl pH 8.0 was added into each aliquot to quench the excessive crosslinking. 10 µl was uploaded into 6% native PAGE, to identify the position of the complex. Fractions containing the complex were selected, and the buffer was changed into 100 mM NaCl and 20 mM HEPES, pH 7.5. As a control, LA 430–579 was prepared in a similar manner. For peptide bound nucleosome complex, 1 mM of the 17-amino-acid peptide (SSGDPAEYNLRSRTVLC, PEPTIDE 2.0 Inc.) was mixed with 1 µM nucleosome in 100 mM NaCl, 20 mM HEPES, pH 7.5 buffer.

Vitrified grids were prepared by applying 3 µl of 1 uM complex sample to a freshly glow-discharged holey carbon grids (Quantifoil R1.2/1.3 Au 200 mesh). The sample was blotted for 3–5 s at 4 °C with 100% humidity and plunged-frozen in liquid nitrogen cooled ethane (FEI™, Vitrobot). Electron micrographs were acquired using a 300 kV Titan Krios G3 (Thermo Fisher Scientific) equipped with a BioQuantom and K3 direct electron detector (Gatan) in super-resolution mode. All images were recorded by EPU (Thermo Fisher Scientific) with a pixel size of 0.65 Å and a defocus range from 0.8 µM to 2.8 µM with a total exposure of ~70 e^−^/Å^2^.

### Cryo-EM data processing

Datasets were processed by CryoSPARC v4.4.1^80^ following the workflow in Extended Data Figs.,7,8, Supplementary Fig. 2. Initially, all movies were subjected to live processing. Patch motion correction and patch CTF estimation were applied to the movies. High-quality micrographs were sorted out based on relative ice thickness and CTF resolution. Subsequently, particles were picked using automated template picking and extracted with a box size of 360 pixels, which were then down-sampled to 180 pixels for following 2D classification.

For the LA 430–585/LA 430–579 bound to nucleosome complex, particles selected after several rounds of 2D classification were used for generating three or two ab initio classes, followed by heterogenous refinement. In the case of the peptide-nucleosome complex, particles were used for generating two ab initio classes. Promising classes were subjected to focused 3D classification based on the Ig-like domain density.

Classes of interest were further processes through homogeneous refinement, non-uniform refinement, reference-based motion correction, another round of non-uniform refinement, local CTF refinement, and non-uniform refinement. The quality of the map was validated by FSC validation and local resolution analysis.

For the visualization of the nucleosome structures in Fig. 3d–f, the flexible Ig-like domain density was masked and the mean local resolution within this mask was determined as 6.8 Å. Accordingly, the Ig-like domain in the structure was low-pass filtered to this resolution. The visualization of the map is a composite between the nucleosome structure and the low-pass filtered Ig-like domain density.

For the peptide-nucleosome structure, the initial model was obtained by fitting a well-solved nucleosome model from Protein Data Bank (PDB) 6ZHX ref. ^81^ into the final map using ChimeraX 1.7^76^. The model of the nucleosome was refined into cryo-EM density using Phenix 1.21.2–5419. The model of peptide was manually built in Coot 0.9.8.95^82^ and refined in PHENIX 1.21.2–5419^83^ iteratively. Visualization of all cryo-EM maps and figure preparation were done by Chimera X1.7^76^.

### The 4 fractions sequential analysis of macromolecules accessibility sequencing, DNA extraction, library preparation and sequencing

The 4f-SAMMY-seq was performed on 5×10^5^ MEFs at 90% confluency using 12 units of DNase I (Invitrogen, AM2222) as described in^43^.

Libraries were then qualitatively and quantitatively checked on and run on the TapeStation System. Libraries with distinct adapter indexes which were normalized to a concentration of 2 nM, equimolarly, pooled, and then loaded onto the Illumina NextSeq 2000 instrument. The sequencing was performed with a minimal target of 15 million reads for 100 bases in single-end mode on the Illumina NextSeq 2000 instrument.

### RNA extraction, library preparation and sequencing

Total RNA was extracted from 1×10^6^ MEFs at 90% of confluence using TRI-Reagent (Sigma, T9424) following the recommended guidelines. The quantification of total RNA was performed with a Qubit 4 fluorometer using the Qubit RNA BR Assay Kit (Invitrogen, Q10210), and RNA integrity was assessed by the Agilent 2100 Bioanalyser with the Agilent RNA 6000 Nano Kit (Agilent, 5067–1511). For each sample, 10 ng of total RNA was used to construct a strand-specific RNAseq library with SMARTer Stranded Total RNA-Seq Kit - Pico Input (Takara, 634487). The quality and the size of the libraries were analyzed with TapeStation System according to the assay guide. The final RNAseq libraries were adjusted to a concentration of 4 nM, equimolarly, pooled, and then loaded onto the Illumina NextSeqTM 550 system. A sequencing depth of 20 million for 75 bases in paired-ends mode was achieved for each sample.

### Chromatin immunoprecipitation sequencing

MEFs were grown to 90% confluence in DMEM (Gibco, 10566-016) supplemented with 10% (v/v) FBS (Gibco,10270106) and then fixed 10 min at RT with 1% formaldehyde (Sigma-Aldrich, F8775), adding 1:10 of formaldehyde solution (50 mM HEPES-KOH pH 7.5, 100 mM NaCl, 1 mM EDTA, 0.5 mM EGTA, and 11% formaldehyde) on cultured cells. Formaldehyde was then quenched with addition of Glycine (Carlo Erba, 453807) to a final concentration of 125 mM for 5 min at RT, followed by two washes with cold 1xPBS. Cross-linked cells were rapidly collected in a falcon tube by scraping on ice and centrifuged at 2000 g at 4 °C. Pellets of 3×10^6^ cells were stored at −80 °C until sonication. Each 3×10^6^ cell pellet was resuspended in 600 μl of cold Lysis Buffer: 50 mM HEPES-KOH, pH 7.5, 10 mM NaCl, 1 mM EDTA, 10% glycerol, 0.5% NP-40, and 0.25% Triton X-100. After 10 min on a rotator at 4 °C, samples were centrifuged for 5 min at 1350 g at 4 °C and resuspended in 130 μl of sonication buffer: 10 mM Tris-HCl pH 8.0, 2 mM EDTA, 1x Protease Inhibitor Cocktail (Roche, 04693116001); 1 mM PMSF (Sigma-Aldrich, 93482) and 0.25% SDS (for histone modifications) or 0,1% SDS (for lamin A/C). Cells were subjected to lysis on ice for 1 h and homogenized by pipetting every 15 min. Total extracted chromatin was sonicated in a Covaris M220 focused-ultrasonicator using snap cap microTUBEs (Covaris, 520045) (water bath set to 7 °C, peak power 75.0, duty factor 10.0, cycles/burst 250, duration 420 s). Fragmentation of chromatin to an average size of 150–500 bp was checked on Agilent 2100 Bioanalyser using High Sensitivity DNA Kit (Agilent, 5067–4626). To reduce the SDS, ChIP of histone marks were dilute to a final concentration 0.1% SDS by adding 1 volume of equilibration buffer: 10 mM Tris-HCl pH 8.0, 233 mM NaCl, 1.66% Triton X-100, 0.166% Deoxycholic acid sodium salt (DOC), 1 mM EDTA, 1x Protease Inhibitor Cocktail (Roche, 04693116001), and 1 mM PMSF (Sigma-Aldrich, 93482). Samples were then centrifuged at 14000 g for 10 min at 4 °C to pellet insoluble material. Supernatants were quantified using Nanodrop 1000 spectrophotometer. For immunoprecipitation, and 80 ug of chromatin was used for each histone modifications and 150 ug for lamin A/C in a final volume of 300 μl of IP buffer: 10 mM Tris-HCl pH8, 140 mM NaCl, 1.66% Triton X-100, 0.166% DOC, 0.1% SDS, 1 mM EDTA, 1x Protease Inhibitor Cocktail (Roche, 04693116001); 1 mM PMSF (Sigma-Aldrich, 93482). 3% of total chromatin was preserved at 4 °C as input normalization-control for each experimental condition. The remaining chromatin was incubated overnight on a rotator at 4 °C with 6 μg of H3K9me3 (Abcam, ab8898) or H3K27ac (Abcam, ab4729) or H3K4me3 (Sigma-Aldrich, 07-473) or 15 μg of lamin A/C (Abcam. ab26300) antibodies. 20 μl of Protein G beads (Life Technology, 1004D) for each IP were washed twice in 0.1% BSA/IP buffer and incubated on rotator overnight at 4 °C. On the next day, protein G beads were added to each sample and incubated on rotator for 2 hours at 4 °C. Beads/IP complexes were then washed 10 min on rotator at 4 °C twice with IP buffer, twice with high-salt IP buffer: 10 mM Tris-HCl pH 8, 500 mM NaCl, 1.66% Triton X-100, 0.166% DOC, 0.1% SDS, 1 mM EDTA, 1x Protease Inhibitor Cocktail (Roche, 04693116001); 1 mM PMSF (Sigma-Aldrich, 93482); twice with RIPA-LiCl buffer: (10 mM Tris-HCl pH 8.0, 1 mM EDTA, 250 mM LiCl, 0.5% DOC, 0.5% NP-40, 1x Protease Inhibitor Cocktail (Roche, 04693116001); 1 mM PMSF (Sigma-Aldrich, 93482); twice with 10 mM Tris-HCl pH 8.0. Crosslinking was reversed by incubating the beads and input at 65 °C overnight with 100 μl of Elution buffer: 10 mM Tris-HCl pH 8, 0,5 mM EDTA, 300 mM, and 0.4% SDS. The next day, all samples were diluted with 100 μl of 1x TE buffer, treated with 2.5 U of RNAse cocktail (Ambion, AM2286) at 37 °C for 120 min, followed by addition of 100 ug of Proteinase K (Invitrogen, AM2548) at 55 °C for 120 min. DNA was then isolated using phenol/chloroform (Sigma-Aldrich, 77617) extraction, followed by a back extraction of phenol/chloroform with additional volume of 1x TE buffer. DNA was precipitated in 2 volumes of cold ethanol, 0.3 M sodium acetate and 20 ug glycogen (Ambion AM9510) for overnight at −20 °C. Pellets were suspended in 31 μl of nuclease-free water and quantified using Qubit 2.0 fluorometer with Qubit dsDNA HS Assay Kits (Invitrogen, Q32854). The libraries were then prepared using the NEBNext Ultra II DNA Library Prep Kit for Illumina (NEB, BE7645L) and NEBNext Multiplex Oligos for Illumina (NEB, BE6440S). Libraries were then qualitatively and quantitatively checked and run on the TapeStation System. Libraries with distinct adapter indexes were normalized to a concentration of 2 nM, equimolarly pooled, and then loaded onto the Illumina NextSeq 2000 instrument. The sequencing was performed with a minimal target of 15 million reads for 100 bases in single-end mode on the Illumina NextSeq 2000 instrument.

### Lamin A construct transfections and fluorescence-activated cell sorting

WT MEFs were transfected with plasmids encoding full-length (LA 1-646) or truncated (LA 1-429) of lamin A C-tagged with eGFP, using Lipofectamine 3000 reagent (Thermo Fisher Scientific, L3000001), according to the manufacturer’s protocol. Briefly, 150,000 cells were seeded in a 6-well plate 12 hours before transfection. The medium was replaced with serum- and antibiotic-free medium, 5 µg of plasmid DNA was combined with 5 µL of Lipofectamine 3000 reagent in OPTIMEM (Thermo Fisher Scientific, 31985062) and added to each well. After 6 hours, the medium was replaced with fresh complete growth medium. eGFP-positive transfected cells were sorted 24 hours post-transfection on FACS Aria SORP (BD), seeded for an additional 24 hours, and then collected for 4f-SAMMY-seq analysis To quantify the percentage of EGFP-positive cells and mean fluorescence intensity (MFI), 50,000 WT cells transfected with pEGFP-LA 1-646 or pEGFP-LA 1-429 were acquired on a BD FACSCanto flow cytometer and analyzed using FlowJo software. Unstained cells were used as negative controls, and sequential gating was performed to exclude debris and doublets.

### DNA sequence analysis

The results of the sequencing were demultiplexed with bcl2fastq (https://support.illumina.com/sequencing/sequencing_software/bcl2fastq-onversion-software.html, v2.19.0.316). Both 4f-SAMMY-seq and ChIP-seq high-throughput sequencing lanes were merged with the help of GNU parallel software (http://www.gnu.org/s/parallel). All sequencing reads were trimmed using Trimmomatic (v0.39)^84^ with clip file ‘TruSeq3-SE-2. fa’ and the following parameters: 2:30:10 for seed mismatch, palindrome threshold and simple threshold, respectively; 4:15 for sliding window. The minimum threshold of 36 bp length was applied for all reads. Trimmed reads were aligned using BWA (v0.7.17-r1188)^85^ setting -n 2 -k 2 parameters and using as reference genome the mm10 version downloaded from refgenie^86^. The result was saved in BAM file format. PCR duplicates were marked and removed with Picard (v2.23.9) (https://github.com/broadinstitute/picard) ‘MarkDuplicates’ option, collecting the filtered reads in another BAM file. All the reads with mapping quality lower than 1 were filtered out with Samtools (v1.11)^87^ creating another BAM file used for subsequent analyses.

### Genomic reads distribution profiles

For each alignment, coverage analyses were performed using Deeptools (v3.5.0)^88^ with ‘bamCoverage’ function. Original trimmed reads of 100 bp were extended up to 250 bp and normalized with RPKM method with a bin size of 50 bp. The mm10 size was considered of 2652783500 bp, as suggested in the Deeptools manual https://deeptools.readthedocs.io/en/latest/content/feature/effectiveGenomeSize.html) and blacklisted regions, known to be problematic in terms of sequencing reads coverage, were obtained by ENCODE portal (https://www.encodeproject.org/files/ENCFF547MET) and excluded from the analysis. To generate the normalized relative enrichment ratios along the genome for each sample using the 4f-SAMMY fractions, the SPP, R package (v1.16.0)^89^ was used and the library was built under R (v4.1.2). The relative enrichment ratio between 4f-SAMMY-seq fractions was calculated per each biological replicate by comparing the S2S reads against the S3 reads, which was used as baseline ($\frac{\rm{S2S}}{\rm{S3}}$). Fraction specific reads were imported from BAM files with ‘read.bam.tags’ function, additionally filtered with ‘remove.local.tag.anomalies’, and the relative differential enrichment was computed using ‘get.smoothed.enrichment.mle’ function setting ‘tag.shift = 0’ and ‘background.density.scaling = TRUE’. The resulting computed signal corresponded to the log2 ratio between the pair of sequencing samples. We defined the solubility profile as the relative enrichment ratio of 4f-SAMMY-seq sequencing reads distribution along the genome for S2S vs S3 fractions. To maximize comparability with 4f-SAMMY-seq data, we computed the normalized relative enrichment ratios of ChIP-seq IP over INPUT genomic profiles using the same methodology described above. To compute correlations between genomic tracks, the smoothed differential signal enrichment was re-binned with Deeptools ‘multiBigwigSummary’ at 50 kb. Genome-wide Spearman correlations between 4f-SAMMY-seq fractions and between 4f-SAMMY-seq solubility profile and ChIP-seq was computed using Deeptools, using the function ‘plotCorrelation’ with the following settings: ‘–corMethod spearman -p heatmap–skipZeros’.

### Enrichment ratio normalization and consensus generation

Each solubility profile was imported with R (v4.3) using the library GenomicRanges (v1.52.0)^90^, resized at genome wide level with 50 kb window. Adjacent blacklisted regions were merged if separated by less than 50 kb with the function ‘reduce’ and setting ‘min.gapwidth= 50000’. It is worth remarking that having the resulting binned genome with a fixed window size of 50 kb, the genomic region adjacent and upstream to the merged blacklist region could be reduced to a different length <50 kb. On the other hand, the genomic region adjacent and downstream to the merged blacklist region were extended to 50 kb, reproducing the original window size. All solubility profiles were normalized by quantile normalization with the preprocessCore library (v1.62.1) (10.18129/B9.BIOC.PREPROCESSCORE) using the function ‘normalize.quantiles’. The consensus track of each group of samples was generated by computing the mean $\bar{x}$ of normalized solubility profiles for each genomic window. The shaded areas represent standard error intervals calculated as ${\rm{SE}}=\,\frac{\sigma }{\sqrt{n}}$ where *σ* is the standard deviation and *n* is the number of samples.

### Genomic track representation

The visualization of genomic tracks was performed with Gviz R library (v 1.44.1)^91^. The ChIP-seq samples were imported using the function ‘import’ of the rtracklayer library and plotted using the function ‘plotTracks’ setting the value ‘windowSize = 1500’ to plot a fine grain profile. Visualization of the 4f-SAMMY-seq consensus tracks were computed using all normalized samples of each group (for example WT, LmnaKO, LBDKO) setting the parameter type as ‘a’ and ‘confint’ overlayed using the function ‘OverlayTrack’. Single samples mountain plots were computed by setting the parameter type as ‘polygon’. Extra elements of the panel, chromosome ideogram and relative genome axis, were displayed using the functions ‘IdeogramTrack’ and ‘GenomeAxisTrack’, respectively. The murine cytobands of the chromosome ideogram were downloaded from the UCSC golden path (http://hgdownload.cse.ucsc.edu/goldenpath/mm10/database/cytoBand.txt.gz).

### Differential enrichment analysis of 4f-SAMMY-seq

Differential enrichment analyses were calculated on consensus tracks. Using the WT solubility profile, all the genomic regions with a solubility value outside the arbitrary threshold of ≤ ±0.1 were filtered out. Accordingly, the LmnaKO solubility profile was used as reference for LmnaKO vs. LBDKO comparison. Each genomic bin with a solubility score >+0.1 was considered euchromatin (S2S > S3), while each genomic bin with a solubility score <−0.1 was considered heterochromatin (S2S < S3). For each comparison (WT vs. LmnaKO, WT vs. LBDKO and LmnaKO vs. LBDKO), considering ± 2 standard error intervals for the reference strain, differentially enriched bins were identified as genomic regions where all solubility scores of the compared strain fell outside the interval of consensus of reference (WT or LmnaKO). Candidate bins were then validated with a two tailed z-test with a confidence interval of 0.99, with the function z.test from BSDA R library (v1.2.2) (10.32614/CRAN.package.BSDA) and adjusted using the Benjamini-Hochberg method for multiple testing correction with the ‘p.adjust’ function and the argument ‘BH’. S2S differentially enriched bins were classified as ‘S2S UP’ or ‘S2S DOWN’ if, with respect to the reference strain, the compared strain increased or decreased its solubility score. S3 differentially enriched bins were classified as ‘S3 UP’ or ‘S3 DOWN’ if, in respect to the reference strain, the compared strain decreased (with more negative numerical values) or increased its solubility score.

### ChIP-seq peak calling

Significant enriched regions of ChIP-seq for the lamin A and H3K9me3 were called in wt MEF, with EDD^92^ (v1.1.19), with configuration options set as: ‘max_CI_value = 0.25’, ‘required_fraction_of_informative_bins = 0.98’, ‘p_hat_CI_method = agresti_coull’ and ‘log_ratio_bin_size = 10’. The options –bin-size and –gap-penalty for the H3K9me3 ChIP-seq were set to 75 for bin-size and 15 for gap-penalty, whereas for lamin A ChIP-seq they were set to 50 for bin-size and 15 for gap-penalty.

### SAMMY-seq and ChIP-seq metaprofile

To compute the average profile over a set of genomic regions of interest (that is, the meta-profile analysis), average enrichment ratio of single H3K9me3 ChIP-seq replica, previously rebinned at resolution of 2 Kb and normalized with quantile normalization with preprocessCore library (v1.62.1) (10.18129/B9.BIOC.PREPROCESSCORE) using the function ‘normalize.quantiles’, was plotted over the S3 UP and S3 DOWN regions, together with 1 Mb of upstream and downstream regions. Each genomic region (upstream, S3 UP or DOWN, downstream) was further divided in 600 subregions with R (v4.3) using the library GenomicRanges (v1.52.0)^90^ and the function ‘tile’. For SAMMY-seq metaprofiles, the same methodology was applied on the enrichment ratios over the H3K9me3 ChIP-seq peaks called with EDD^92^ (v1.1.19). The mean signal of IP over the INPUT or the SAMMY-seq enrichment ratios were measured in each tile for all the regions of the metaprofile with the function ‘mean’ and the ChIP-seq signal was smoothed by ggplot2 (https://ggplot2.tidyverse.org) with the function ‘geom_smooth’ using method = ‘gam’ and formula = ‘y ~ s (x, bs = “cs”)’. To statistically compute the difference of H3K9me3 signal at the peak’s borders, each H3K9me3 domain was partitioned into 200 equal-sized bins subregions with R (v4.3) using the library GenomicRanges (v1.52.0) with the function ‘tile’. For each sample, the mean signal intensity of IP over the INPUT within each bin was calculated. The B point was defined as the bin located in the centre of domain. The A point was defined as the bin corresponding to the Domain Start (DS). Then, the A point value was subtracted from the B point value, yielding the B-A difference for each peak within each sample. Finally, a two-sided, unpaired Wilcoxon rank test was performed using the ‘wilcox.test’ function from the ‘stats’ base R package (version 4.3.1), with the ‘alternative’ parameter set to ‘two.sided’ and the ‘paired’ parameter set to ‘FALSE’.

### RNA-seq analysis

Sequenced reads were analyzed with the pipeline nf-core/rnaseq version^93^. The overall quality of the sequenced reads was assessed using FastQC 2 (v0.11.9) (https://www.bioinformatics.babraham.ac.uk/projects/fastqc/). Reads were trimmed and adapters were clipped by cutadapt (v3.4) (10.14806/ej.17.1.200). Reads overlapping ribosomal RNA (rRNA) were filtered with SortMeRNA^94^ (v4.3.4), considering all the available databases of rRNA. Reads were then mapped in paired end mode with STAR (v2.7.10a)^95^ on the mouse genome mm10 version. Transcripts were quantified using Salmon^96^ on GENCODE (M25)^75^. Basic gene annotation filtered for transcript with HAVANA characteristics (https://www.sanger.ac.uk/project/manual-annotation/#:~:text=The%20HAVANA%20team%20manually%20annotate,well%20as%20poly%2Dadenylation%20features) and protein coding genes. The full matrix with gene level quantification was created by importing transcript abundances with R (v4.3) library tximport (v1.28.0)^97^ using the function ‘tximport’ and setting the parameter ‘type = “salmon”’. The resulting matrix loaded in R (v4.3) was constructed with genes with a total count greater than 15 in all conditions. The counts were normalized with DESeq2 (v1.40.2), using the median of ratios^98^ and accounting for batch effect using the ‘design = ~ batch + condition’. RNA-seq samples were grouped in different flow cell batches as follows: Batch 1 contained replicates 3 for LmnaKO and 3 for WT. Batch 2 included replicates 2 for WT, 1-2 for LmnaKO, and 1-2-3 for LBDKO. Finally, Batch 3 consisted solely of replicate 4 of the WT. The differential expression test was done using the default Wald Test and Benjamini and Hochberg correction for multiple tests, to compute p-values and adjusted p-values, respectively. Differentially expressed genes (DEGs) were identified as those with an adjusted p-value < 0.05 and the absolute value of the | Log2 Fold Change | > 3. Because of high p-values in differential expression results, we also applied the ‘lfcShrink’ function of DESeq2 with type ‘ashr’^99^. The volcano plot showing the DEGs have been done with EnhancedVolcano library (v1.18.0) (10.18129/B9.bioc.EnhancedVolcano).

### RNA-seq metaprofile

To compute the metaprofile of the solubility score at differentially expressed genes, the solubility profiles previously rebinned at a resolution of 2 kb and normalized using the preprocessCore library (v1.62.1) (10.18129/B9.BIOC.PREPROCESSCORE) using the function ‘normalize.quantiles’. The plots were made using Deeptools (v3.5.0). The merging distance between blacklisted regions was fixed at 5 kb. The 4f-SAMMY-seq solubility score was computed per DEG using the command ‘computeMatrix’ from deeptools scale-regions with setting ‘–beforeRegionStartLength 5000–regionBodyLength 5000–afterRegionStartLength 5000–skipZeros’. The metaprofile was plotted using the command ‘plotProfile’ with the specific settings–plotType heatmap–yMin −0.25–yMax 0.75’.

### Chromatin compartments analysis

Chromatin compartments were calculated using a revised version of the CALDER algorithm (version 1.0)^100^ as implemented in the original 4f-SAMMY-seq pipeline^43^. Namely, for each chromosome: i) the four 4f-SAMMY-seq fractions’ (S2S, S2L, S3, and S4) reads distribution profiles were calculated and normalized with RPKM using Deeptools (v3.5.0), rebinned at 50 kb and merged blacklisted regions were filtered out using R (v4.3); ii) for each genomic bin, defined as a vector containing the four RPKM values (one for each fraction), the Euclidean distance (dist, R stats package, method = ‘euclidean’) was calculated with all the other bins of the same chromosome. These steps produced an NxN matrix, where N is the number of bins for the considered chromosome. Starting from this matrix, the eigenvector was derived to reconstruct chromatin compartmentalization. Sub-compartment segmentation was limited to the highest level, thus obtaining only 2 compartments corresponding to ‘A’ and ‘B’ compartments. The compartment analysis was performed independently for each chromosome. The consensus of compartment calls across strains was produced by labeling each genomic bin (50 kb) according to the most recurring call across samples: for example, if a bin is labeled as A in more than half of the WT samples, that bin will be defined as A in the consensus of controls. Bins with an equal assignment of compartments were not considered in the consensus. The definition of compartment shifts was based on the concordant or discordant compartment classification with respect to WT for each 50 kb genomic bin. In the comparison between LMNA1-646 and LMNA1-429 transfected cells, we assessed A-to-B and B-to-A compartment changes using Cohen’s *d* effect size analysis. For each genomic region, we extracted the corresponding eigenvalue signals across biological replicates and calculated effect sizes using the cohen.d() function from the *effsize* R package (v1.16.0). Our analysis revealed that over 70% of regions undergoing compartment switches exhibited medium to large effect sizes (*d* ≥ 0.5 and *d* ≥ 0.8, respectively).

### Gene Ontology

GO analysis for both RNA-seq differential expression analysis and compartment switches analysis were performed for Biological Process – BP and Molecular Function – MF using R (v4.3) with gprofiler2 library (v0.2.2) (10.32614/CRAN.package.gprofiler2) and the GO database id ‘e111_eg58_p18_b51d8f08’, taking as final enriched terms only those with p-value < 0.01, calculated by the function ‘gost’ with correction_method = ‘g_SCS’ (Set Counts and Sizes)^101^.

### Primers

Lmna-Bamh1-394-F: cgcggatccacctcgcagcgcagcc; Lmna-Bamh1-430-F: cgcggatccttctcacagcacgcacgcact; Lmna-Sal1-548-R: acgcgtcgacttaagtcactgagcgcaccagc; Lmna-Sal1-560-R: acgcgtcgacttagtcatctccatcctcatc; Lmna-Sal1-579-R: acgcgtcgacttagtactcagcggggtccc; Lmna-Sal1-585-R: acgcgtcgacttaggtgcgcgagcgcaggttgt; Lmna-Sal1-646-R: acgcgtcgacttagtaggagcgggtgaccag; Psumo-Bamh1-Lmnb1-432-F: acagattggtggatccatctctcattccgcctca; Psumo-Sal1-Lmnb1-569-R: ccgcaagcttgtcgacttaaagttcttcctcaacaac; Pegfp-N1-Lmna-Nhe1-F: tttagtgaaccgtcagatccgctagcgccaccatggttgagaccccgtcccagcgg; Pegfp-N1-Lmna-Ecor1-R: ccgcggtaccgtcgactgcacgaattcgtaggagcgggtgaccagat; Pegfp-N1-Lmna-429-Ecor1-R: ccgcggtaccgtcgactgcacgaattcgctgctgcggctctcagt.

### Reporting summary

Further information on research design is available in the Nature Portfolio Reporting Summary linked to this article.

## Online content

Any methods, additional references, Nature Portfolio reporting summaries, source data, extended data, supplementary information, acknowledgements, peer review information; details of author contributions and competing interests; and statements of data and code availability are available at 10.1038/s41594-025-01622-5.

## Supplementary information

Supplementary Information Supplementary Figs. 1 and 2.

Reporting Summary

Supplementary Video 1 Tomogram of the NE region of a WT MEF. This tomogram of the NE region of a WT MEF is shown in Extended Data Fig. 1a as a single xy-slice. The complete tomogram is shown.

Supplementary Video 2 Tomogram of the NE region of a LmnaKO MEF. This tomogram of the NE region of a LmnaKO MEF is shown in Extended Data Fig. 1b as a single xy-slice. The complete tomogram is shown.

Supplementary Video 3 Tomogram of the NE region of a LBDKO MEF. This tomogram of the NE region of a LBDKO MEF is shown in Extended Data Fig. 1c as a single xy-slice. The complete tomogram is shown.

## Source data

Source Data Statistical source data for all main and extended data figures.

Source Data Fig. 3 Unprocessed western blots and/or gels.

Source Data Extended Data Fig. 6 Unprocessed western blots and/or gels.

Source Data Extended Data Fig. 7 Unprocessed western blots and/or gels.

## Data Availability

The cellular tomograms and sub-tomogram averaging structures were deposited in the EMDB: EMD-19827, EMD-19828, EMD-19829, EMD-52630, and EMD-52633. The density map of lamin A 430–585 with nucleosome was deposited in EMDB: EMD-50291. The complex structure of lamin A 572–588 with nucleosome was deposited in the EMDB (EMD-50114) and PDB (PDB-9F0O). The high-throughput sequencing data generated for this study are available in the NCBI GEO database with the following accession numbers: ‘GSE268922, GSE268923, GSE268924’. Source data have been provided in Source data. All other data supporting the findings of this study are available from the corresponding authors on reasonable request. Source data are provided with this paper.
