# Supplementary material for: The molecular basis of lamin-specific chromatin interactions
Source: Nat Struct Mol Biol. 2025 Aug 1;32(10):1999–2011. doi: 10.1038/s41594-025-01622-5 (PMC12527912; doi:10.1038/s41594-025-01622-5)
Supplement: Supplementary file 1 — Supplementary Figs. 1 and 2. [file 41594_2025_1622_MOESM1_ESM.pdf]

nature structural & molecular biology

Supplementary information

https://doi.org/10.1038/s41594-025-01622-5

The molecular basis of lamin-specific chromatin interactions

In the format provided by the authors and unedited

Supplementary Information

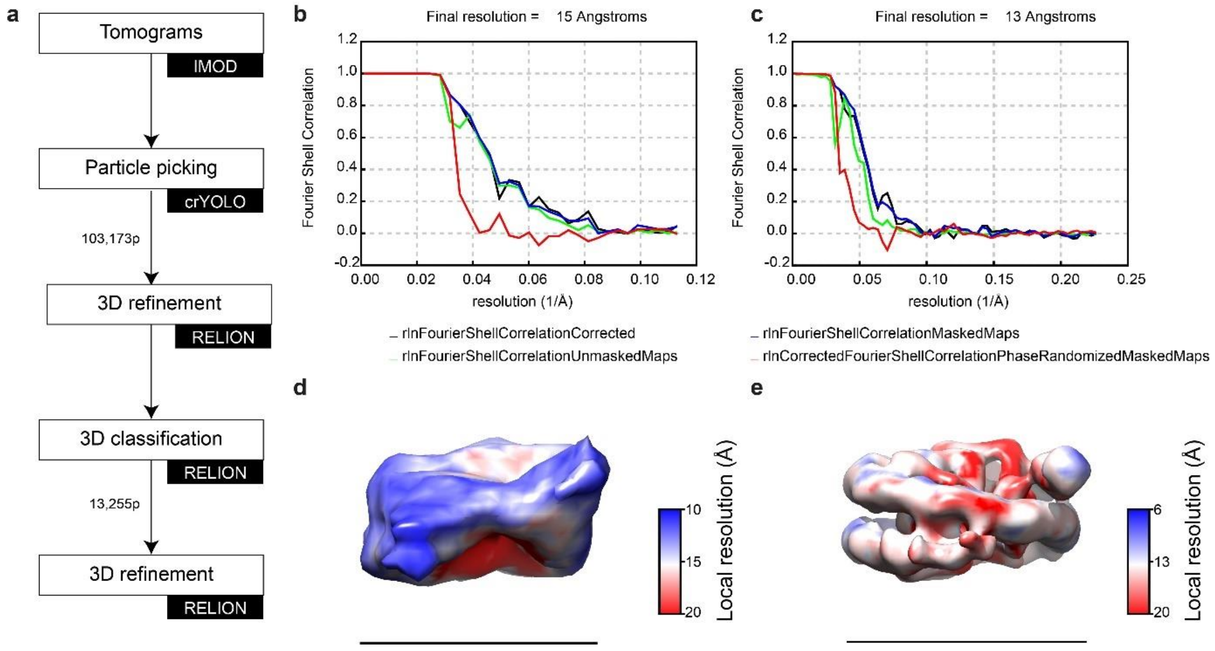

Supplementary Figure 1. Structural analysis of in-situ nucleosomes. a. Flowchart illustrating the in-situ nucleosome processing pipeline. **b**. and **c**. FSC curves for the in-situ nucleosome consensus average and for the in-situ nucleosome canonical average. Resolution is measured at the 0.143 criterion. **d**. and **e**. Local resolution maps of the in-situ nucleosome consensus average and of the in-situ nucleosome canonical average. Scalebar is 10nm.

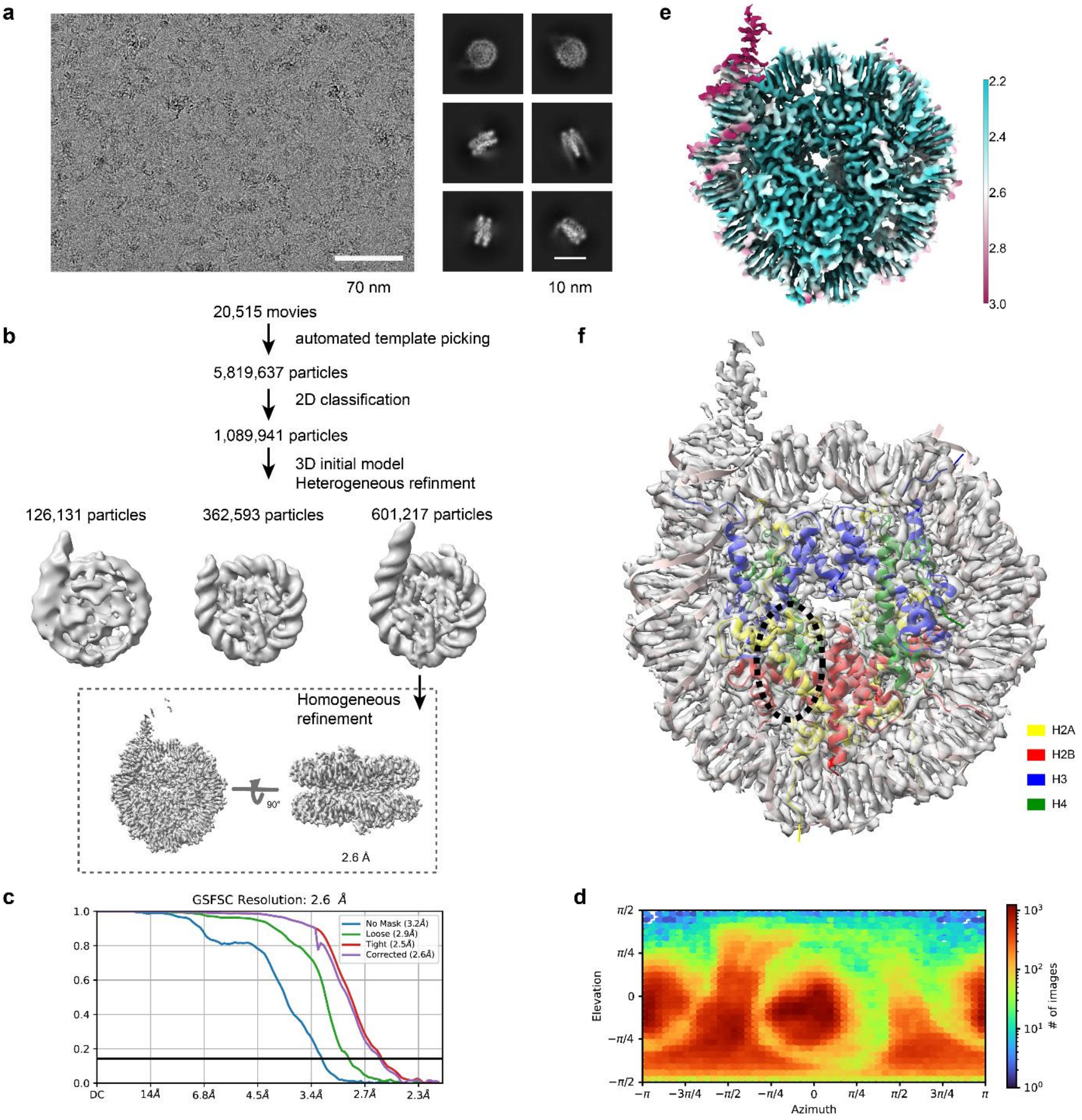

Supplementary Figure 2. Structural analysis of LA 430-579-nucleosome complex structure. a. Representative cryo-EM image and 2D class averages of LA 430-579-nucleosome complex. **b.** Flow chart for image processing by cryoSPARC. **c.** FSC curve for final 3D map (2.6Å). Resolution is given for the FSC 0.143 criterion. **d.** Angular distribution of particles. A calibration bar is provided. **e.** Local resolution of final reconstruction. **f.** The final 3D map fitted by nucleosome structure shown there is no more additional density near the acidic patch (black dashed circle).
