# Supplementary material for: The molecular basis of lamin-specific chromatin interactions
Source: Nat Struct Mol Biol. 2025 Aug 1;32(10):1999–2011. doi: 10.1038/s41594-025-01622-5 (PMC12527912; doi:10.1038/s41594-025-01622-5)
Supplement: Supplementary file 2 — Reporting Summary [file 41594_2025_1622_MOESM2_ESM.pdf]

nature portfolio

Corresponding author(s): Chiara Lanzuolo and Ohad Medalia

Last updated by author(s): May 28, 2025

Reporting Summary

Nature Portfolio wishes to improve the reproducibility of the work that we publish. This form provides structure for consistency and transparency in reporting. For further information on Nature Portfolio policies, see our Editorial Policies and the Editorial Policy Checklist.

Statistics

For all statistical analyses, confirm that the following items are present in the figure legend, table legend, main text, or Methods section.

n/a	Confirmed	
☐	☒	The exact sample size (*n*) for each experimental group/condition, given as a discrete number and unit of measurement
☐	☒	A statement on whether measurements were taken from distinct samples or whether the same sample was measured repeatedly
☐	☒	The statistical test(s) used AND whether they are one- or two-sided<br>*Only common tests should be described solely by name; describe more complex techniques in the Methods section.*
☒	☐	A description of all covariates tested
☐	☒	A description of any assumptions or corrections, such as tests of normality and adjustment for multiple comparisons
☐	☒	A full description of the statistical parameters including central tendency (e.g. means) or other basic estimates (e.g. regression coefficient) AND variation (e.g. standard deviation) or associated estimates of uncertainty (e.g. confidence intervals)
☐	☒	For null hypothesis testing, the test statistic (e.g. *F*, *t*, *r*) with confidence intervals, effect sizes, degrees of freedom and *P* value noted<br>*Give P values as exact values whenever suitable.*
☒	☐	For Bayesian analysis, information on the choice of priors and Markov chain Monte Carlo settings
☒	☐	For hierarchical and complex designs, identification of the appropriate level for tests and full reporting of outcomes
☒	☐	Estimates of effect sizes (e.g. Cohen's *d*, Pearson's *r*), indicating how they were calculated

Our web collection on statistics for biologists contains articles on many of the points above.

Software and code

Policy information about availability of computer code

Data collection	SerialEM (3.4.9, 3.8.0, 3.9.0), EPU (2.14),BD FACSCanto

Data analysis

MATLAB (R2019b)
TOM toolbox(2.x)
IMOD (4.9.12)
MotionCor2 (1.4.0)
Gctf (1.06)
crYOLO (1.8.2)
RELION(4.0.1,5.0)
UCSF ChimeraX(1.7)
AreTomo (1.3.3)
cryoSPARC (4.4.1)
Coot(0.9.8.95)
Phenix(1.21.2-5419)
FlowJo™ (v10.10)
R (v4.1)
R (v4.3)
Prism (v10.1.1)
GraphPad Prism 10
Origin 2018

For manuscripts utilizing custom algorithms or software that are central to the research but not yet described in published literature, software must be made available to editors and reviewers. We strongly encourage code deposition in a community repository (e.g. GitHub). See the Nature Portfolio guidelines for submitting code & software for further information.

Data

Policy information about availability of data

All manuscripts must include a data availability statement. This statement should provide the following information, where applicable:

- Accession codes, unique identifiers, or web links for publicly available datasets
- A description of any restrictions on data availability
- For clinical datasets or third party data, please ensure that the statement adheres to our policy

The cellular tomograms and sub-tomogram averaging structures were deposited in the EMDB: EMD-19827, EMD-19828, EMD-19829, EMD-52630, and EMD-52633. The density map of lamin A 430-585 with nucleosome was deposited in EMDB: EMD-50291. The complex structure of lamin A 572-588 with nucleosome was deposited in the EMDB: EMD-50114 and PDB: 9F0O. The high-throughput sequencing data generated for this study are available in the NCBI GEO database with following accession numbers 'GSE268922, GSE268923, GSE268924'. Source data have been provided in Source Data. All other processed data supporting the findings of this study are available from the corresponding author on reasonable request.

Human research participants

Policy information about studies involving human research participants and Sex and Gender in Research.

Reporting on sex and gender	N/A
Population characteristics	N/A
Recruitment	N/A
Ethics oversight	N/A

Note that full information on the approval of the study protocol must also be provided in the manuscript.

Field-specific reporting

Please select the one below that is the best fit for your research. If you are not sure, read the appropriate sections before making your selection.

☒ Life sciences ☐ Behavioural & social sciences ☐ Ecological, evolutionary & environmental sciences

For a reference copy of the document with all sections, see nature.com/documents/nr-reporting-summary-flat.pdf

Life sciences study design

All studies must disclose on these points even when the disclosure is negative.

Sample size

150 tomograms were acquired for the cryo-FIB/cryo-ET analysis from 3 different cell types,
>140,000 subtomograms of nucleosomes were analyzed from cells. (Rafael Kronenberg-Tenga, et al. 2021 https://doi.org/10.1242/jcs.256156)

> 200,000 nucleosome images were used for every structure
500,000 cells were used for SAMMY-seq(Federica Lucini, et al.2024 https://doi.org/10.1093/nar/gkae454)
3,000,000 cells were used for ChIP-seq(Landt, S.G., et al. 2012. https://doi.org/10.1101/gr.136184.111)
50,000 cells were used for transfection efficiency analysis
1,000,000 cells where used for RNA-seq (Conesa, A., et al. 2016 https://doi.org/10.1186/s13059-016-0881-8)

Data exclusions	None
Replication	At least 3 independent replications were performed for all EM measurements.<br>3 independent replicates were used for RNA-seq and 4f-SAMMY-seq high-throughput sequencing technology<br>2 independent replicates were used for ChIP-seq high-throughput sequencing technology<br>All attempts to replicate the results were successful.
Randomization	Randomization was not applicable in this study because each experimental group was explicitly associated with a specific MEF or lamin KO cell line. The comparisons were made between predefined biological groups (i.e., distinct cell lines), not randomly assigned conditions.
Blinding	Blinding was not performed in this study because the experimental design involved the use of three distinct MEF cell lines, each representing a specific experimental group. Data collection and analysis were conducted with consistent protocols across all groups to minimize bias.

Reporting for specific materials, systems and methods

We require information from authors about some types of materials, experimental systems and methods used in many studies. Here, indicate whether each material, system or method listed is relevant to your study. If you are not sure if a list item applies to your research, read the appropriate section before selecting a response.

Materials & experimental systems

n/a	Involved in the study	
☐	☒	Antibodies
☐	☒	Eukaryotic cell lines
☒	☐	Palaeontology and archaeology
☒	☐	Animals and other organisms
☒	☐	Clinical data
☒	☐	Dual use research of concern

Methods

n/a	Involved in the study	
☐	☒	ChIP-seq
☐	☒	Flow cytometry
☒	☐	MRI-based neuroimaging

Antibodies

Antibodies used	For 80 µg of chromatin, 6 µg of H3K9me3 (Abcam, ab8898), 6 µg of H3K27ac (Abcam, ab4729), 6 µg of H3K4me3 (Sigma-Aldrich, 07-473), or 15 µg of Lamin A/C (Abcam, ab26300) were used.
Validation	Anti-Histone H3 (tri methyl K9) (Abcam, ab8898)<br>Validated for ChIP, ICC/IF, IHC-P, WB on human and mouse. Specific for H3K9me3 with minor cross-reactivity for H3K27me3. Supported by 1,737 publications. Enrichment profile: GSE268923_MEFwt1_H3K9me3vsInput_mle.bw.<br>Anti-Histone H3 (acetyl K27) (Abcam, ab4729)<br>Validated for ChIP, ICC/IF, IHC-P, WB on human, mouse, rat, and cow. Specific for H3K27ac. Supported by 2,917 publications. Enrichment profile: GSE268923_MEFwt1_H3K27acvsInput_mle.bw.<br>Anti-Histone H3 (tri methyl K4) (Sigma-Aldrich, 07-473)<br>Validated for ChIP, ChIP-seq, WB, dot blot, and ICC on human, mouse, and rat. Specific for H3K4me3. Supported by 1,164 publications. Enrichment profile: GSE268923_MEFwt1_H3K4me3vsInput_mle.bw.<br>Anti-Lamin A/C (Abcam, ab26300)<br>Validated for ChIP, ICC/IF, IHC-P, and WB on human, mouse, and rat. Specific for Lamin A/C proteins. Supported by 117 publications. Enrichment profile: GSE268923_newMEFwt_laminAvsinput_mle.bw.

Eukaryotic cell lines

Policy information about cell lines and Sex and Gender in Research

Cell line source(s)	Wildtype, DKOLMNB and LmnaKO mouse embryonic fibroblast (MEF) were kindly provided by Y. Zheng and R.D. Goldman. Published in: https://www.nature.com/articles/cr2013118 and https://doi.org/10.1091/mbc.E15-07-0461
Authentication	PCR assays using species-specific primers were performed to authenticate the identity of each MEF cell line.
Mycoplasma contamination	The cell line tested negative for mycoplasma contamination.
Commonly misidentified lines (See ICLAC register)	No commonly misidentified cell lines were used in the study.

ChIP-seq

Data deposition

☒ Confirm that both raw and final processed data have been deposited in a public database such as GEO.

☒ Confirm that you have deposited or provided access to graph files (e.g. BED files) for the called peaks.

Data access links
May remain private before publication.

https://www.ncbi.nlm.nih.gov/geo/query/acc.cgi?acc=GSE268922 secure token krszogkgrlcplcv
https://www.ncbi.nlm.nih.gov/geo/query/acc.cgi?acc=GSE268923 secure token qhwlkeiezzqnrur

https://www.ncbi.nlm.nih.gov/geo/query/acc.cgi?acc=GSE268924 secure token idgdioiofbsrfej

Files in database submission

GSE268922 code with GSM samples
GSM8302711 RNA,MEFLMNACKO,rep1
GSM8302712 RNA,MEFLMNBDKO,rep1
GSM8302713 RNA,MEFLMNACKO,rep2
GSM8302714 RNA,MEFLMNBDKO,rep2
GSM8302715 RNA,MEFwt,rep2
GSM8302716 RNA,MEFLMNACKO,rep3
GSM8302717 RNA,MEFLMNBDKO,rep3
GSM8302718 RNA,MEFwt,rep3
GSM8302719 RNA,MEFwt,rep4

GSE268923 code with GSM samples
GSM8302720 ChIP,MEFACKO,rep1,H3K9me3
GSM8302721 ChIP,MEFACKO,rep1,Input
GSM8302722 ChIP,MEFACKO,rep2,H3K9me3
GSM8302723 ChIP,MEFACKO,rep2,Input
GSM8302724 ChIP,MEFBDKO,rep1,H3K9me3
GSM8302725 ChIP,MEFBDKO,rep1,Input
GSM8302726 ChIP,MEFBDKO,rep2,H3K9me3
GSM8302727 ChIP,MEFBDKO,rep2,Input
GSM8302728 ChIP,MEFwt,rep1,H3K27ac
GSM8302729 ChIP,MEFwt,rep1,H3K4me3
GSM8302730 ChIP,MEFwt,rep1,H3K9me3
GSM8302731 ChIP,MEFwt,rep1,Input
GSM8302732 ChIP,MEFwt,rep2,H3K9me3
GSM8302733 ChIP,MEFwt,rep2,Input

GSE268924 code with GSM samples
GSM8302734 SAMMY,MEFACKO,rep1,S2L
GSM8302735 SAMMY,MEFACKO,rep1,S2S
GSM8302736 SAMMY,MEFACKO,rep1,S3
GSM8302737 SAMMY,MEFACKO,rep1,S4
GSM8302738 SAMMY,MEFBDKO,rep1,S2L
GSM8302739 SAMMY,MEFBDKO,rep1,S2S
GSM8302740 SAMMY,MEFBDKO,rep1,S3
GSM8302741 SAMMY,MEFBDKO,rep1,S4
GSM8302742 SAMMY,MEFwt,rep1,S2L
GSM8302743 SAMMY,MEFwt,rep1,S2S
GSM8302744 SAMMY,MEFwt,rep1,S3
GSM8302745 SAMMY,MEFwt,rep1,S4
GSM8302746 SAMMY,MEFACKO,rep2,S2S
GSM8302747 SAMMY,MEFACKO,rep2,S2L
GSM8302748 SAMMY,MEFACKO,rep2,S3
GSM8302749 SAMMY,MEFACKO,rep2,S4
GSM8302750 SAMMY,MEFBDKO,rep2,S2L
GSM8302751 SAMMY,MEFBDKO,rep2,S2S
GSM8302752 SAMMY,MEFBDKO,rep2,S3
GSM8302753 SAMMY,MEFBDKO,rep2,S4
GSM8302754 SAMMY,MEFACKO,rep3,S2L
GSM8302755 SAMMY,MEFACKO,rep3,S2S
GSM8302756 SAMMY,MEFACKO,rep3,S3
GSM8302757 SAMMY,MEFACKO,rep3,S4
GSM8302758 SAMMY,MEFBDKO,rep3,S2L
GSM8302759 SAMMY,MEFBDKO,rep3,S2S
GSM8302760 SAMMY,MEFBDKO,rep3,S3
GSM8302761 SAMMY,MEFBDKO,rep3,S4
GSM8302762 SAMMY,MEFwt,rep3,S2L
GSM8302763 SAMMY,MEFwt,rep3,S2S
GSM8302764 SAMMY,MEFwt,rep3,S3
GSM8302765 SAMMY,MEFwt,rep3,S4
GSM8302766 SAMMY,MEFwt,rep2,S2L

GSM8302767 SAMMY,MEFwt,rep2,S2S
GSM8302768 SAMMY,MEFwt,rep2,S3
GSM8302769 SAMMY,MEFwt,rep2,S4

GSE268923: Add the following GSM samples:
GSM8833390 – ChIP, MEFwtlamin, rep1, LaminAC_IP
GSM8833391 – ChIP, MEFwtlamin, rep1, Input
GSE268924: Add the following GSM samples:
GSM8837962 – SAMMY-seq, MEFwtLamA, rep0, S2L
GSM8837963 – SAMMY-seq, MEFwtLamA, rep0, S2S
GSM8837964 – SAMMY-seq, MEFwtLamA, rep0, S3
GSM8837965 – SAMMY-seq, MEFwtLamA, rep0, S4
GSM8837966 – SAMMY-seq, MEFwtLamAtrunc, rep0, S2L
GSM8837967 – SAMMY-seq, MEFwtLamAtrunc, rep0, S2S
GSM8837968 – SAMMY-seq, MEFwtLamAtrunc, rep0, S3
GSM8837969 – SAMMY-seq, MEFwtLamAtrunc, rep0, S4
GSM8837970 – SAMMY-seq, MEFwtLamA, rep1, S2L
GSM8837971 – SAMMY-seq, MEFwtLamA, rep1, S2S
GSM8837972 – SAMMY-seq, MEFwtLamA, rep1, S3
GSM8837973 – SAMMY-seq, MEFwtLamA, rep1, S4
GSM8837974 – SAMMY-seq, MEFwtLamAtrunc, rep1, S2L
GSM8837975 – SAMMY-seq, MEFwtLamAtrunc, rep1, S2S
GSM8837976 – SAMMY-seq, MEFwtLamAtrunc, rep1, S3
GSM8837977 – SAMMY-seq, MEFwtLamAtrunc, rep1, S4
GSM8837978 – SAMMY-seq, MEFwtLamAtrunc, rep2, S2L
GSM8837979 – SAMMY-seq, MEFwtLamAtrunc, rep2, S2S
GSM8837980 – SAMMY-seq, MEFwtLamAtrunc, rep2, S3
GSM8837981 – SAMMY-seq, MEFwtLamAtrunc, rep2, S4
GSM8837982 – SAMMY-seq, MEFwtLamA, rep3, S2L
GSM8837983 – SAMMY-seq, MEFwtLamA, rep3, S2S
GSM8837984 – SAMMY-seq, MEFwtLamA, rep3, S3
GSM8837985 – SAMMY-seq, MEFwtLamA, rep3, S4
GSM8837986 – SAMMY-seq, MEFwtLamAtrunc, rep3, S2L
GSM8837987 – SAMMY-seq, MEFwtLamAtrunc, rep3, S2S
GSM8837988 – SAMMY-seq, MEFwtLamAtrunc, rep3, S3
GSM8837989 – SAMMY-seq, MEFwtLamAtrunc, rep3, S4

Genome browser session (e.g. UCSC)

Provide a link to an anonymized genome browser session for "Initial submission" and "Revised version" documents only, to enable peer review. Write "no longer applicable" for "Final submission" documents.

Methodology

Replicates

4f-SAMMY-seq and RNA-seq were done on 3 distinct replicates for each mutants and wild-type. H3K9me3 ChIP-seq were done on 2 distinct replicates for each mutants and wild-type. H3K27ac and H3K4me3 were done on 2 distinct replicates in the wild-type. Lamin A/C ChIP-seq was done on 1 replica (Abcam, ab26300).

Sequencing depth

The sequencing was performed with a minimal target of 15 million reads for 100 bases in single-end mode
A sequencing depth of 20 million for 75 bases in paired-ends mode was achieved for RNA-seq samples

Antibodies

H3K9me3 (Abcam, ab8898) ,H3K27ac (Abcam, ab4729),H3K4me3 (Sigma-Aldrich, 07-473), lamin A/C (Abcam, ab26300)

Peak calling parameters

Specify the command line program and parameters used for read mapping and peak calling, including the ChIP, control and index files used.

Data quality

Describe the methods used to ensure data quality in full detail, including how many peaks are at FDR 5% and above 5-fold enrichment.

Software

Describe the software used to collect and analyze the ChIP-seq data. For custom code that has been deposited into a community repository, provide accession details.

Flow Cytometry

Plots

Confirm that:

☒ The axis labels state the marker and fluorochrome used (e.g. CD4-FITC).

☒ The axis scales are clearly visible. Include numbers along axes only for bottom left plot of group (a 'group' is an analysis of identical markers).

☒ All plots are contour plots with outliers or pseudocolor plots.

☒ A numerical value for number of cells or percentage (with statistics) is provided.

Methodology

Sample preparation	50,000 cells were collected, rinsed with 1X PBS, and resuspended in 200 µL of 1X PBS before acquisition.
Instrument	BD FACSCanto II flow cytometer system
Software	FlowJo™ v10.10
Cell population abundance	At least 15,000 events were acquired.
Gating strategy	Forward and side scatter gating (FSC-A vs SSC-A) were used to identify cells and remove debris. Gating for single cells (FSC-A vs FSC-H). Gating for EGFP-positive (EGFP+) transfected cells based on Alexa Fluor 488-A fluorescence intensity, using gates defined from untransfected samples. The resulting percentage of cells were plotted using Prism.

☒ Tick this box to confirm that a figure exemplifying the gating strategy is provided in the Supplementary Information.
