# Supplementary material for: The molecular basis of lamin-specific chromatin interactions
Source: Nat Struct Mol Biol. 2025 Aug 1;32(10):1999–2011. doi: 10.1038/s41594-025-01622-5 (PMC12527912; doi:10.1038/s41594-025-01622-5)
Supplement: Supplementary file 7 — Unprocessed western blots and/or gels. [file 41594_2025_1622_MOESM7_ESM.pdf]

Figure 3c

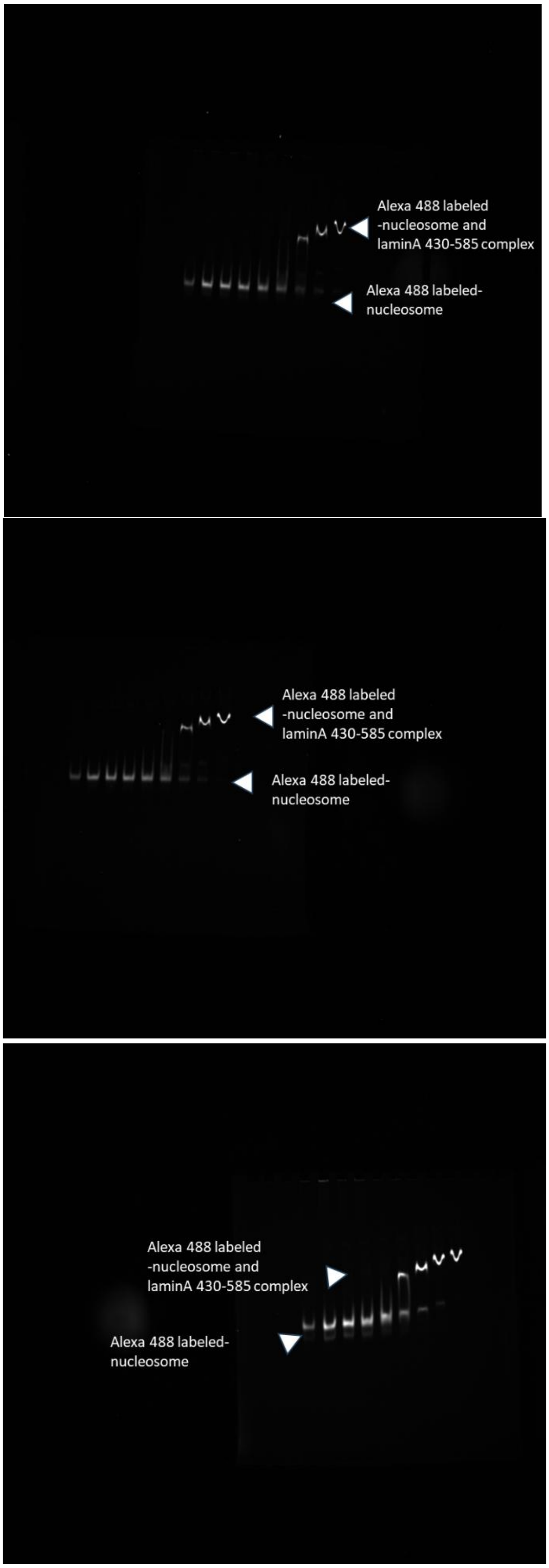
Alexa 488 labeled
-nucleosome and
laminA 430-585 complex
Alexa 488 labeled-
nucleosome
Alexa 488 labeled
-nucleosome and
laminA 430-585 complex
Alexa 488 labeled-
nucleosome
Alexa 488 labeled
-nucleosome and
laminA 430-585 complex
Alexa 488 labeled-
nucleosome
