# Supplementary figures and images for: The molecular basis of lamin-specific chromatin interactions
Source: Nat Struct Mol Biol. 2025 Aug 1;32(10):1999–2011. doi: 10.1038/s41594-025-01622-5 (PMC12527912; doi:10.1038/s41594-025-01622-5)

Extended Data Fig. 6b

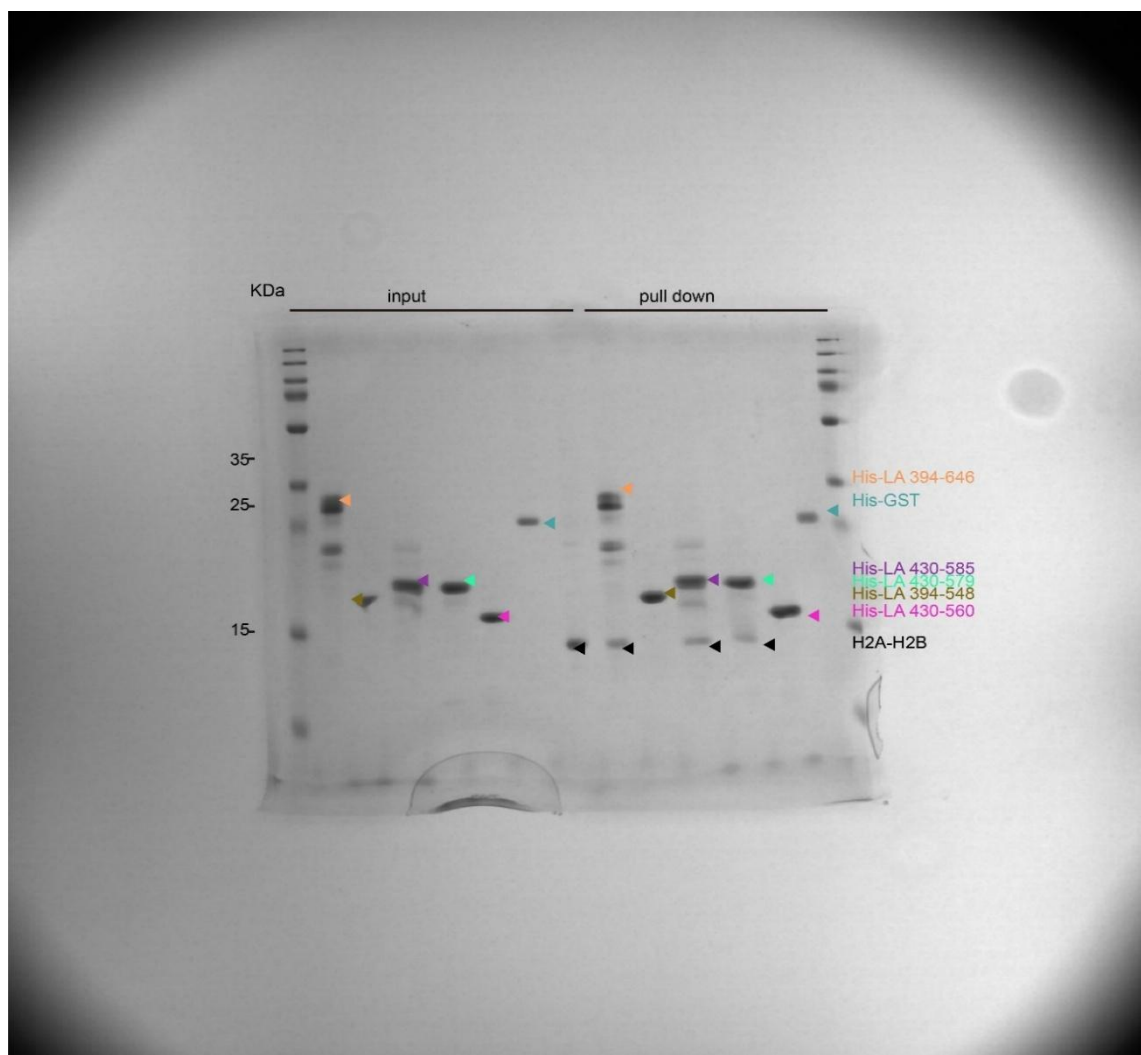

Supplement: Supplementary file 8 — Unprocessed western blots and/or gels. [file 41594_2025_1622_MOESM8_ESM.pdf]

Extended Data Fig. 7a

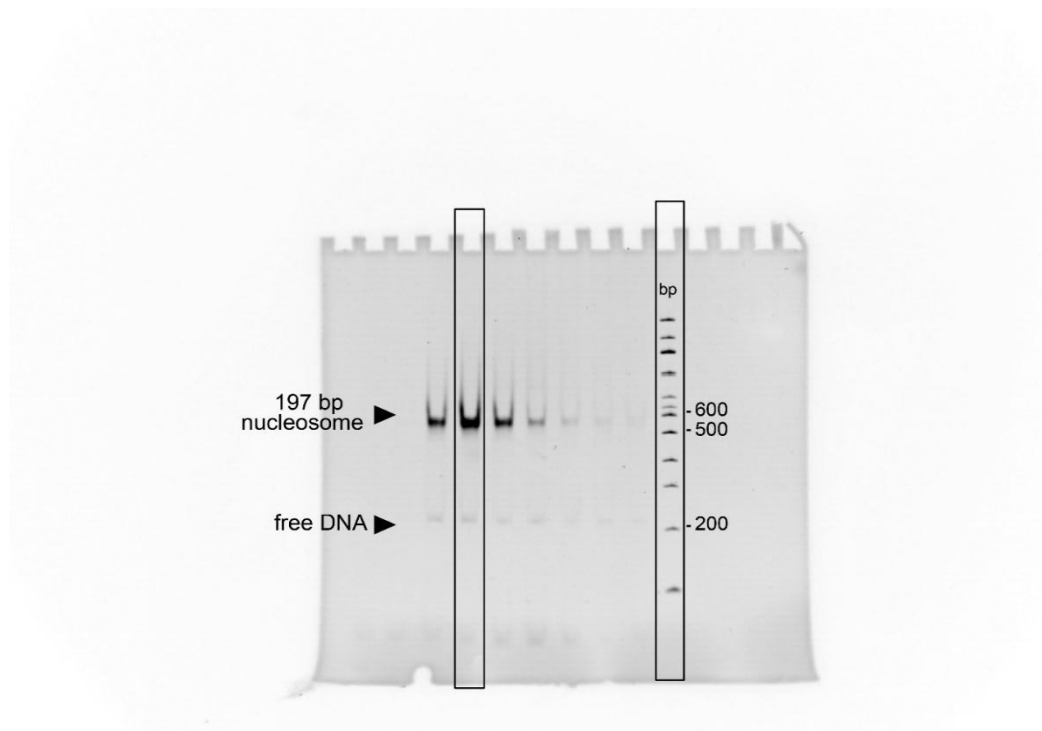

Extended Data Fig. 7b

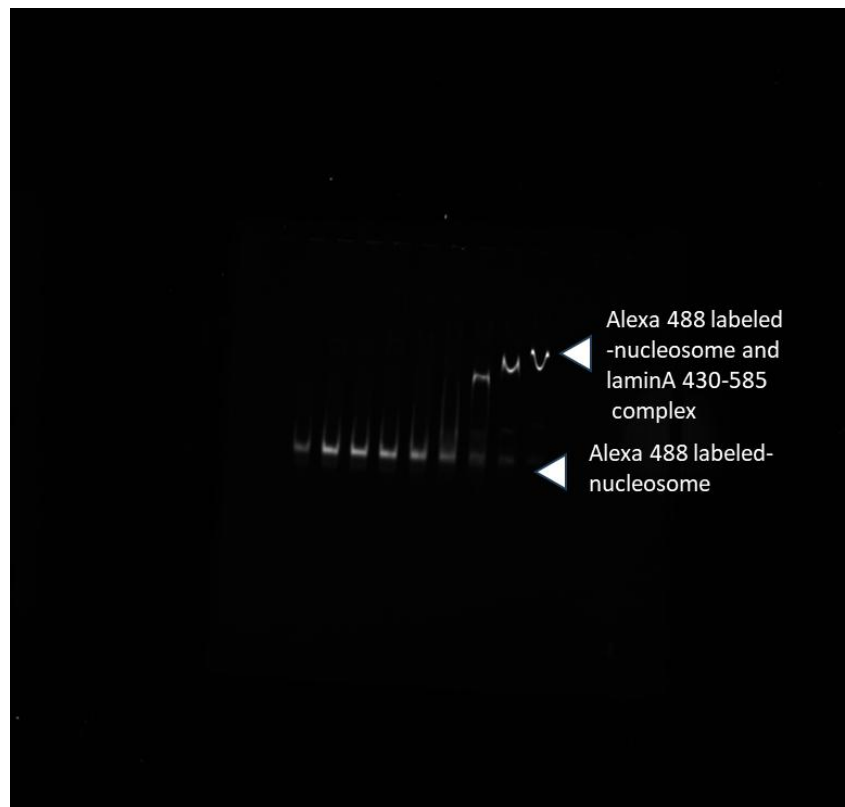

Extended Data Fig. 7b

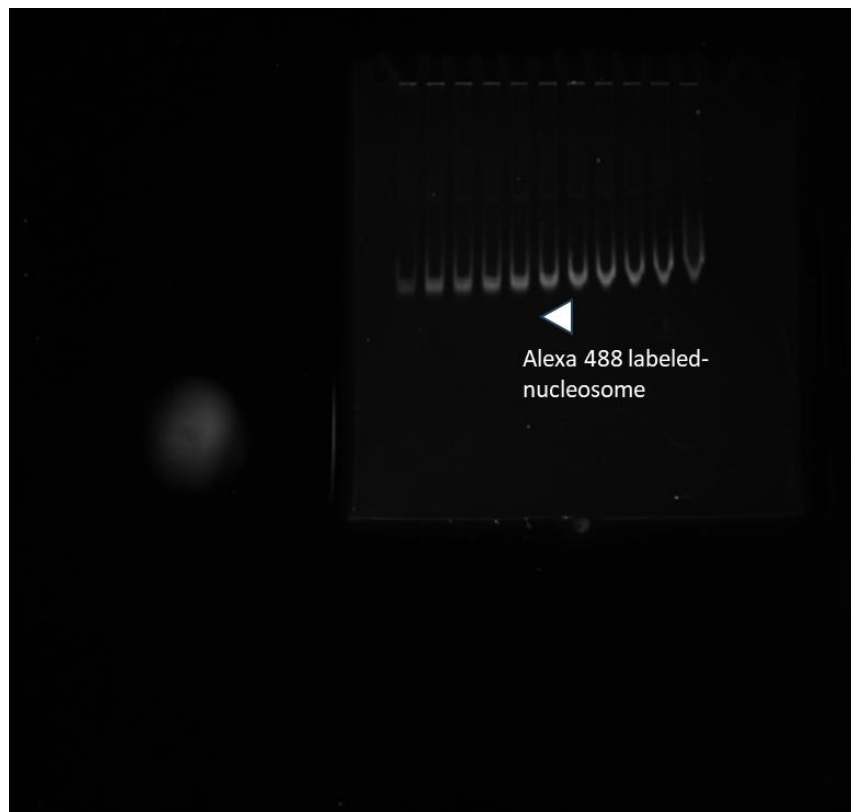

Extended Data Fig. 7d

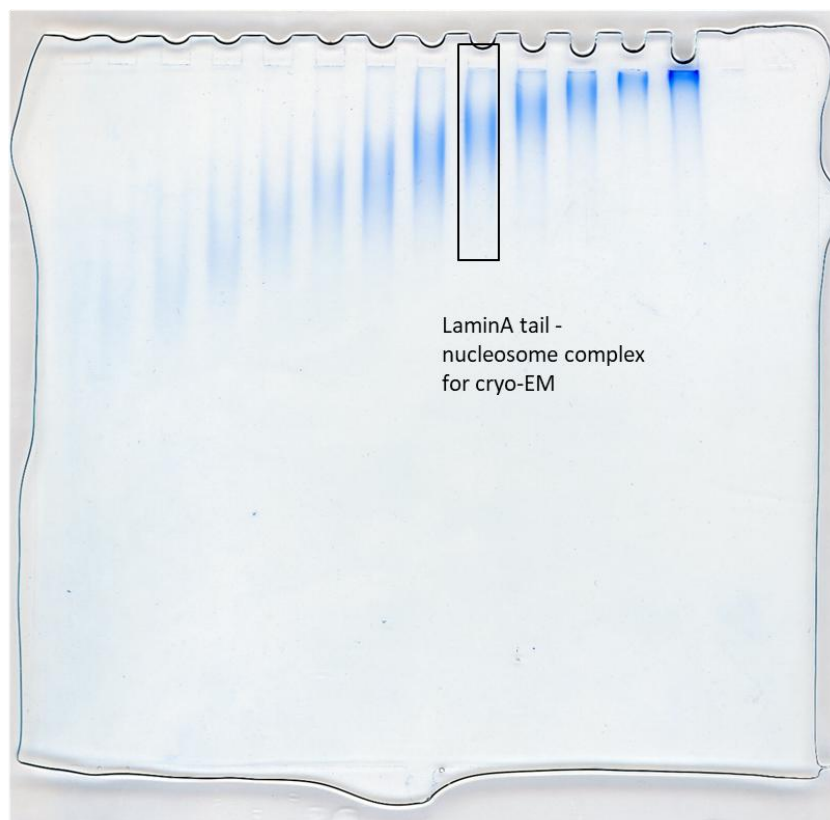

Supplement: Supplementary file 9 — Unprocessed western blots and/or gels. [file 41594_2025_1622_MOESM9_ESM.pdf]
